# *Major histocompatibility complex *(*Mhc*) class Ib gene duplications, organization and expression patterns in mouse strain C57BL/6

**DOI:** 10.1186/1471-2164-9-178

**Published:** 2008-04-17

**Authors:** Masato Ohtsuka, Hidetoshi Inoko, Jerzy K Kulski, Shinichi Yoshimura

**Affiliations:** 1 Division of Basic Molecular Science and Molecular Medicine, School of Medicine, Tokai University, Bohseidai, Isehara, Kanagawa 259-1193, Japan; 2 Centre for Forensic Science, The University of Western Australia, Nedlands, WA, Australia

## Abstract

**Background:**

The mouse has more than 30 *Major histocompatibility complex *(*Mhc*) class Ib genes, most of which exist in the *H2 *region of chromosome 17 in distinct gene clusters. Although recent progress in *Mhc *research has revealed the unique roles of several *Mhc *class Ib genes in the immune and non-immune systems, the functions of many class Ib genes have still to be elucidated. To better understand the roles of class Ib molecules, we have characterized their gene duplication, organization and expression patterns within the *H2 *region of the mouse strain C57BL/6.

**Results:**

The genomic organization of the *H2-Q*, -*T *and -*M *regions was analyzed and 21 transcribed *Mhc *class Ib genes were identified within these regions. Dot-plot and phylogenetic analyses implied that the genes were generated by monogenic and/or multigenic duplicated events. To investigate the adult tissue, embryonic and placental expressions of these genes, we performed RT-PCR gene expression profiling using gene-specific primers. Both tissue-wide and tissue-specific gene expression patterns were obtained that suggest that the variations in the gene expression may depend on the genomic location of the duplicated genes as well as locus specific mechanisms. The genes located in the *H2-T *region at the centromeric end of the cluster were expressed more widely than those at the telomeric end, which showed tissue-restricted expression in spite of nucleotide sequence similarities among gene paralogs.

**Conclusion:**

Duplicated *Mhc *class Ib genes located in the *H2-Q*, -*T *and -*M *regions are differentially expressed in a variety of developing and adult tissues. Our findings form the basis for further functional validation studies of the *Mhc *class Ib gene expression profiles in specific tissues, such as the brain. The duplicated gene expression results in combination with the genome analysis suggest the possibility of long-range regulation of *H2-T *gene expression and/or important, but as yet unidentified nucleotide changes in the promoter or enhancer regions of the genes. Since the *Mhc *genomic region has diversified among mouse strains, it should be a useful model region for comparative analyses of the relationships between duplicated gene organization, evolution and the regulation of expression patterns.

## Background

The *Major Histocompatibility Complex *(*MHC*) genomic region harbors duplicated genes that express protein molecules responsible for the rejection of transplanted tissue, restricted antigen presentation and the recognition of self and non-self [1,2]. The *Mhc *genomic region in the mouse, located on chromosome 17, is named *H2 *and the genes within this region are usually classified into three distinct classes (I to III) based on their structure and function [3]. The class I molecules generally elicit immune responses by presenting peptide antigens derived from intracellular proteins to T lymphocytes and their genes can be classified into two groups, the classical *Mhc *class I (class Ia) genes and the non-classical *Mhc *class I (class Ib) genes. The classical *Mhc *class Ia genes, such as *H2-K *and -*D *in the mouse, are highly polymorphic, expressed widely and present antigens to CD8+ cytotoxic T cells. To date, most studies of the *MHC *class I genomic region have been focused on the immunological function of class Ia molecules [4-6].

The non-classical class Ib molecules are structurally similar to the classical class Ia proteins, but in contrast to the classical class Ia proteins, they have limited or no polymorphisms. They are more restricted in their tissue expression and some have functions other than antigen presentation to CD8+ T cells. The non-classical class Ib proteins have shorter cytoplasmic tails and some of them lack consensus residues associated with peptide binding [7]. The mouse is considered to have more than 30 *Mhc *class Ib genes in the genome [3]. Most *Mhc *class Ib genes are located at the telometric end of the 2 Mb-*H2 *region within the *H2-Q*, -*T *and -*M *sub-regions, which were originally mapped and defined by recombination analysis. Although the non-classical class Ib genes are involved in immunological functions like the classical class Ia genes, they generally serve a more specialized role in the immune responses. The expression and function of some non-classical class Ib genes, including *H2-T23 *(Qa-1), -*M3 *and -*T3 *(TL antigen), have been analyzed in detail. For example, Qa-1 is involved in the suppression of CD4+ T cell responses via CD94/NKG2A or CD94/NKG2C receptors [8,9]. The peptide presentation by the Qa-1 molecule may also have a role in CD8+ regulatory T cell activity [10]. H2-M3 molecules prime the rapid response of CD8+ T cells by presenting *N*-formylated bacterial peptides [11]. The TL antigen is involved in the formation of memory CD8+ T cells [12] and in the regulation of iIEL responses in the intestine by interaction with homodimeric CD8 alpha receptors [13].

The class Ib molecules are also involved in non-immune functions. For example, the *H2-M1 *and -*M10 *families of the class Ib genes specifically interact with the V2R class of pheromone receptors presented on the cell surfaces of the vomeronasal organ [14,15]. The Qa-2 proteins encoded by *H2-Q7 *and -*Q9 *class Ib genes influence the rate of preimplantation embryonic development and subsequent embryonic survival [16]. In addition, the class I molecules have recently been shown to contribute to the development and plasticity of the brain [17,18]. So far, there is little information about which of the non-classical class Ib genes are involved in this function.

The molecular functions of many of the other class Ib molecules are still far from being understood and even the expression patterns for many of the *Mhc *class Ib genes remain to be elucidated. The *Mhc *class Ib genes are members of gene clusters that have been generated by different rounds of duplication and deletion [19]. In the mouse, the telomeric 1 Mb of the *Mhc *including the *H2-M *region was well characterized using the 129/Sv inbred strain [20]. The possible evolutionary fates of duplicated genes are nonfunctionalization, neofunctionalization or subfunctionalization [21]. Genes recently duplicated may even have the same functions by having and using identical or similar expression domain sequences. In order to better understand the role of class Ib molecules expressed by duplicated genes in different tissues, we have undertaken to examine, identify and characterize the *Mhc *class Ib gene duplication, organization and expression patterns within the *H2 *region of the mouse strain C57BL/6.

The whole genome of the laboratory mouse strain C57BL/6J has been almost fully sequenced [22]. However, the genomic organization of the *Mhc *class I region of mice varies markedly between different haplotypes and inbred strains [20]. In the present study, we selected *Mhc *class Ib DNA sequences from the mouse genome database (NCBI Entrez Genome Project ID 9559), and characterized the organization of the *Mhc *class Ib genomic region for the mouse C57BL/6 strain (haplotype b). Expression patterns of each of the *Mhc *class Ib genes were examined by RT-PCR using gene-specific primer sets, and we identified *Mhc *class Ib genes with either tissue-restricted expression or tissue-wide expression. We also identified monogenic and multigenic duplicated regions within the *H2-T *region of the mouse inbred-strain, C57BL/6. Based on the results of our comprehensive analysis of the *Mhc *class Ib gene duplication, organization and expression patterns, we discuss the possible relationships and regulatory outcomes between the genomic location and expression patterns of the mouse *Mhc *class Ib duplicated genes.

## Results and Discussion

### Identification and genomic organization of transcribed Mhc class Ib genes

**Figure 1:**
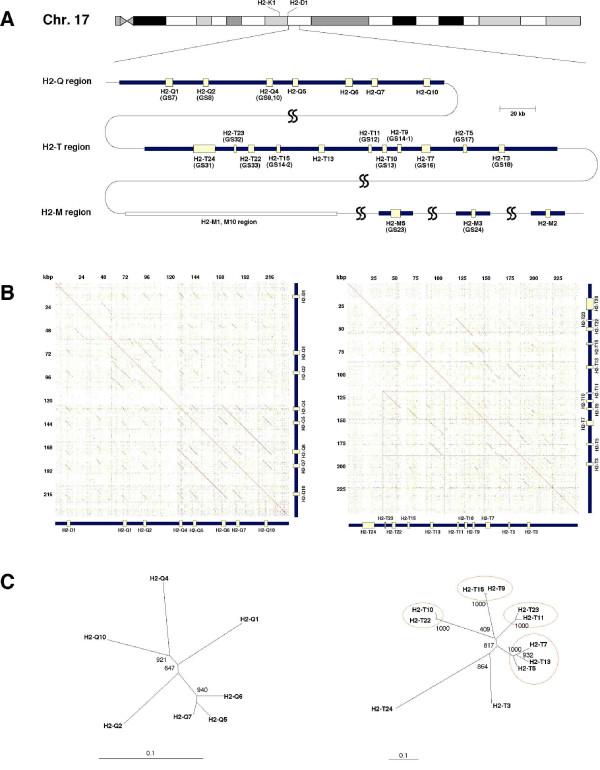
**Genomic organization of the *H2-Q*, -*T *and -*M *region**. (A) Gene content of the *H2-Q*, -*T *and -*M *regions on chromosome 17 of mouse C57BL/6 strain. The *Mhc *class Ib genes with coding potential are represented by yellow boxes. The genes determined by our initial analysis using GENSCAN program are indicated with a GS number. Gene contents regarding *H2-M1 *and -*M10 *families were omitted in this figure. The regions indicated by the squiggles are the regions where the non-*Mhc *genes are interspersed. The scale bar indicating 20 kb applies to the *H2-Q *and -*T *regions. (B) Dot-plot comparisons of the mouse *H2-Q *(left) and -*T *(right) regions. Comparison of the sequence to itself reveals the duplicated regions. (C) Phylogenetic tree analysis of *H2-Q *(left) and -*T *(right) genes based on nucleotide sequences of the entire coding region. Gene pairs showing highly similar sequences (>85%) in *H2-T *region are represented by red circles. Bootstrap values (1000 replicates) are indicated. A scale bar of "0.1" represents a branch-length of 0.1 nucleotide substitutions per site.

As the aim of this study was to determine the tissue expression patterns for each of the duplicated *Mhc *class Ib genes, we first needed to identify the location and the number of transcribing *Mhc *class Ib genes in the mouse genomic sequence [22]. Although a nearly complete mouse genomic sequence of this region was available in the public database, there were many large sequence gaps and incomplete annotations for the sequence when we started this study. Therefore, we predicted the putative *Mhc *class Ib genes from the genomic contig NT_039650.2 by using the GENSCAN program. This analysis identified 19 *Mhc *class Ib-like sequences with coding potential (data not shown). Based on these sequences and the information obtained from the public databases, we designed gene-specific primer sets (Table 1) and confirmed the expression of the predicted genes by RT-PCR against a panel of cDNA tissues as described below. The nucleotide sequences, determined by direct-sequencing of the RT-PCR-amplified fragments, were registered with the GenBank/DDBJ sequence database and given the accession numbers, [GenBank:AB266872, AB266873, AB267092–AB267096]. As a result, a total of 15 expressed genes were identified and mapped onto the current genomic sequence ("GS" number in Figure 1). Although there may be a possibility of misassemblies or missequencing of genomic sequence, most of the assembled sequence, especially the order of genes, is thought to be correct considering the fact that the distributions of restriction sites (such as *Eco*RI, *Bam*HI and *Kpn*I) are consistent with previous reports (data not shown) [23,24], and that the cDNA sequences we examined were perfectly matched with genomic sequence. However, there was no genomic sequence corresponding to the *H2-Q8 *and -*Q9 *genes that are believed to be present in C57BL/10 (haplotype b). At present, we do not know with certainty whether the assembly of the genome is completely correct in this region. Although the *H2-Q5 *locus was annotated as *H2-Q8 *in the genome database, we designated this locus as *H2-Q5 *for the following reasons. 1) This locus was consistent with the physical map position of *H2-Q5 *in the previous report [23], and 2) the DNA sequence of this locus is different from the *H2-Q8 *gene of C57BL/10 (U57392). This analysis in combination with a previous report [25] revealed that a total of at least 21 *Mhc *class Ib genes, 7 in the *H2-Q *region, 11 in the *H2-T *region and 3 in the *H2-M *region are definitely transcribed in the C57BL/6 mouse. However, in the present study, we did not consider the *H2-M1*, -*10 *family of *Mhc *class Ib genes that are located outside the *H2-Q *and -*T *genomic regions.

**Table 1:** List of gene specific primer sets used for expression pattern analysis

Gene	Primer set		Size (bp)
		
	Forward	Reverse	
*H2*-*K1*	GGGAGCCCCGGTACATGGAA	GGTGACTTTATCTTCAGGTCTGCT	548
*H2*-*D1*	TCGGCTATGTGGACAACAAGG	GGCCATAGCTCCAAGGACAC	818
*H2*-*Q1*	CTGCGGTATTTCGAGACCTCG	GGTATCTGTGGAGCCACATCAG	502/686
*H2*-*Q2*	ACACACAGGTCTCCAAGGAA	TGGATCTTGAGCGTAGTCTCTTA	785
*H2*-*Q4*	CTTGCTGAGTTATTTCTACACCT	ACCGTCAGATCTGTGGTGACAT	583
*H2*-*Q5*	GGGAGCCCCGGTTCATCATC	CAGGGTGACAGCATCATAAGATA	539
*H2*-*Q6*	GTATTTCCACACTGCTGTGTCCT	AAGGACAACCAGAATAGCTACGT	871
*H2*-*Q7*	CGGGCCAACACTCGCTGCAA	GTATCTGCGGAGCGACTGCAT	515
*H2*-*Q10*	CACACTCCATGAGGTATTTCGAA	CAGATCAGCAATGTGTGACATGATA	590/866
*H2*-*T24*	ATGCACAGTACTTCACTCATG	CCCCTAGCATATACTCCTGTCG	736/839
*H2*-*T23*	AGTATTGGGAGCGGGAGACTT	AGCACCTCAGGGTGACTTCAT	438
*H2*-*T22*	CTGGAGCAGGAGGAAGCAGATA	CAAATGATGAACAAAATGAAAACCA	698
*H2*-*T15*	ACCGCCCTGGCCCCGACCCAA	CATCCGTGCATATCCTGGATT	332
*H2*-*T13*	GCCCTGACTATGATCGAGACT	CACCTCAGGGTGACATCACCTG	635
*H2*-*T11*	CGGTATTTCCACACCGTCGTA	TAGAGATATGCGAGGCTAAGTTG	415/628
*H2*-*T10*	CCCTTTGGGTTCACACTCGCTT	CCTGGTCTCCACAAACTCCACTTCT	661
*H2*-*T9*	ACCGCCCTGGCCCCGACCCGA	CATCCGTGCATATCCTGGATA	332
*H2*-*T7*	CTTCACACGTTCCAGCTGTTGTT	AGGCCTGGTCTCCACAAGCTCT	432
*H2*-*T5*	GGTGGTGTTGCAGAGACGCT	CTGCTCTTCAACACAAAAGG	482
*H2*-*T3*	TTCAACAGCTCAGGGGAGACTG	AAGCTCCGTGTCCTGAATCAAT	585
*H2*-*M3*	CAGCGCTGTGATAGCATTGA	ACAACAATAGTGATCACACCT	806
*H2*-*M2*	GAGGAGACCCACTACATGACTGTT	GAAAATGAAAGACTGAGGAGGTCTAC	798
b2m*	ATGGCTCGCTCGGTGACCCTG	ATTGCTCAGCTATCTAGGATA	546
GAPDH*	TGAAGGTCGGTGTGAACGGATTTG	GGCCTTCTCCATGGTGGTGAAGAC	314

*Sequences of these primers were obtained from a previous report [25].

Table 2 presents the *H2 *gene numbering system for C57BL/6 mice (haplotype b) that we have used in this paper. We designated each *H2 *gene with reference to the genomic locations and designations used by others [23,24,26]. The nucleotide sequences were determined for the full-length cDNAs expressed by the genes *H2-T23*, -*T22*, -*T15*, -*T5 *and -*M5 *and submitted to the GenBank database [GenBank:AB359227–AB359231]. All genes exhibited a standard class I structure with an alpha 1, alpha 2, alpha 3 and transmembrane (TM) domain.

**Table 2:** List of mouse MHC class Ib genes analysed in this study

Gene name		mRNA sequences referred		
	
Used in this study	Others	NCBI accession	Ensembl transcript ID	NCBI accessions determined in this study
Q1 (GS7)		U96752	ENSMUST00000073208	-
Q2 (GS8)		AY989880	ENSMUST00000074806	AB266872
Q4 (GS9,10)	Qb-1	XR_034205	ENSMUST00000113887	AB267092, AB266873
Q5		-	ENSMUST00000040240	-
Q6		NM_207648	ENSMUST00000091611	-
Q7	Qa-2, Ped	NM_010394	ENSMUST00000071951	-
Q10		AK131620	ENSMUST00000068291	-
T24 (GS31)		NM_008207	ENSMUST00000066488	-
T23 (GS32)	Qa-1		ENSMUST00000102678	AB359230
T22 (GS33)		AK133985	ENSMUST00000058801	AB359229
T15 (GS14-2)		-	ENSMUST00000113742	AB359227
T13	Bl, blastocyst MHC, T25	AY989821	ENSMUST00000025333	-
T11 (GS12)		XM_975970	ENSMUST00000079918	-
T10 (GS13)		NM_010395	ENSMUST00000074201	-
T9 (GS14-1)		-	-	AB267093
T7 (GS16)		NM_001025208	ENSMUST00000064686	-
T5 (GS17)		NM_001081032	ENSMUST00000040467	AB359231
T3 (GS18)	TL	AK033602	ENSMUST00000025312	-
M5 (GS23)	CRW2	XM_903477	ENSMUST00000113667	AB359228
M3 (GS24)	Hmt	NM_013819	ENSMUST00000038580	AB267096
M2	Thy19.4	AY302212	ENSMUST00000077662	-

Haplotype b (C57BL/6) was used. Nomenclature of each gene was based on the previous reports [23, 26], except for GS number. The H2 prefixes were omitted. The GS numbers in parenthesis represent the gene sequence numbers used in our laboratory.

### Monogenic and multigenic duplications

**Figure 2:**
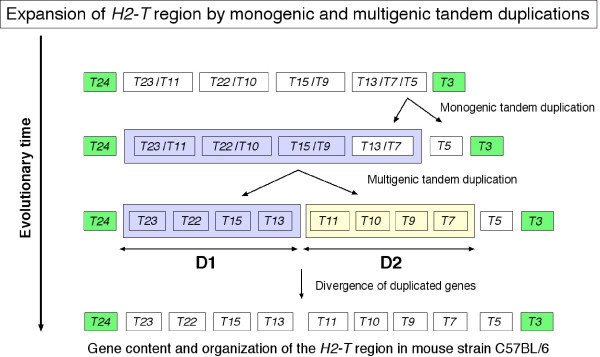
**A schematic model of the expansion of the *H2-T *region by monogenic and multigenic tandem duplications**. This model represents monogenic and multigenic tandem duplications originating from a hypothetical ancestral *H2-T *genomic sequence consisting of six *H2-T *genes. Each labeled box represents a *H2-T *gene in a linear array (horizontal) at different evolutionary times along the vertical axis. The horizontal double arrows labeled D1 and D2 represent the genomic products of the multigenic tandem duplication, with each product consisting of four genes.

**Figure 3:**
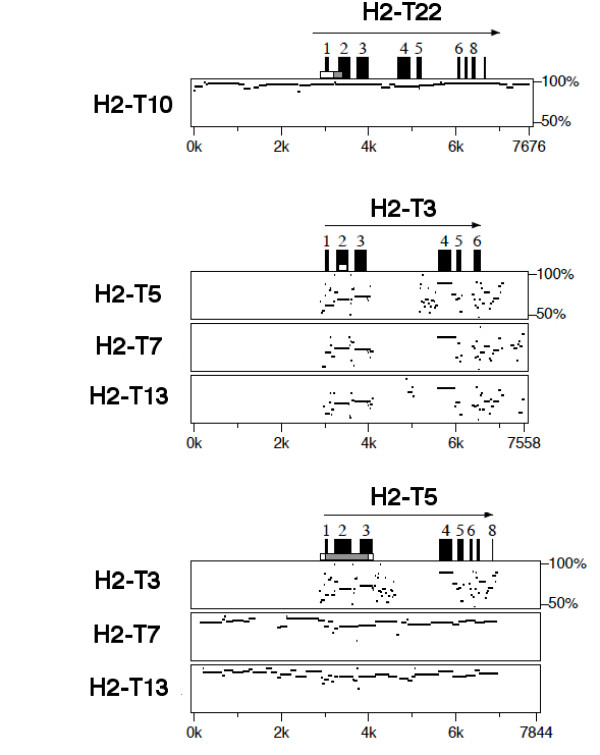
**PipMaker analyses of genomic sequences of mouse *H2-T *genes**. PipMaker analyses were performed to detect similarity within the promoter region. Sequences used for comparison include 3 kb of 5' upstream region and 1 kb of 3' downstream region of coding sequence for each gene. Exons are indicated by black boxes above the plot.

*Mhc *class I genes tend to diversify between species or strains as a result of local duplications and deletions [27]. As local duplication often generates similar genes with similar expression pattern and functional redundancy, it is important to understand the genomic organization and evolution of the *Mhc *class Ib regions. Hence, dot-plot analysis was conducted by comparing the sequences of the *H2-Q *and -*T *regions to themselves (Figure 1B; 240,000 bp for *H2-Q *cluster, 250,000 bp for *H2-T *cluster). In addition to the short diagonal lines seen in the dot-plots due to the similarity of each *Mhc *class I gene, long diagonal lines that indicate evidence of local duplications are seen in both the *H2-Q *and -*T *regions. Regarding the *H2-Q *region, duplication is evident in approximately a 52-kb region from *H2-Q4 *to -*Q10 *(Figure 1B left). A long diagonal line is also seen in the *H2-T *region (Figure 1B right) indicating a multigenic duplication event within the *H2-T *region from *H2-T23 *to -*T5 *(Figure 2). The phylogenetic tree of *H2-T *genes (Figure 1C) supports the occurrence of a multigenic duplication event that produced some gene sets with a high sequence similarity (> 85% in coding region, e.g. *H2-T11 *and -*T23*, *H2-T9 *and -*T15*, *H2-T10 *and -*T22*, and *H2-T5*, -*T7 *and -*T13*). Similarities between these genes are seen not only in the coding region, but also in the untranslated region, introns and intergenic regions (Figures 1B and 3), indicating the possibility that these genes have a redundant function and/or expression pattern.

Figure 2 shows a schematic representation of a single multigenic tandem duplication of four ancestral genes that generated eight genes within the genomic D1 and D2 duplication products. The model also shows that before the occurrence of the multigenic duplication event a single monogenic tandem duplication had probably generated a copy of the *H2-T5 *gene. This parsimonious model helps to explain the gene organization (Figure 1B), phylogenetic topologies of the gene sequences (Figure 1C) and the sequence similarities (Figure 3) between *H2-T23 *and -*T11*, *H2-T22 *and -*T10*, *H2-T15 *and -*T9*, and *H2-T13 *and -*T7*. However, the multigenic duplication model presented here for the mouse *H2-T *region has not taken into account the presence of pseudogenes *T1 *and *T2 *and other evolutionary mechanisms that may have contributed to diversity within this region, such as gene conversions and unequal cross-overs with other haplotypes. Nevertheless, the multigenic duplication model for the mouse *H2-T *region is similar to the multigenic tandem duplication models previously proposed for the *Mhc *class I region of human and non-human primates [28,29].

Regarding the *H2-Q *region, the genes *H2-Q5*, -*Q6 *and -*Q7*, which form a tandem array in the *H2-Q *region (Figure 1B), also grouped relatively closely together in the phylogenetic tree analysis (Figure 1C). Assuming the current genome assembly is correct, then these three genes were probably generated by two separate monogenic tandem duplications much more recently than the duplications previously involved with the generation of the *H2-Q1*, -*Q2*, -*Q4 *and -*Q10 *genes, which are more distantly related in sequence in the phylogenetic analysis. However, the duplication structure of the *H2-Q *region in C57BL/6 (Figure 1B left) appears to be different to the mouse strain 129/SvJ [30].

### Expression of Mhc class Ib genes in adult tissues

**Figure 4:**
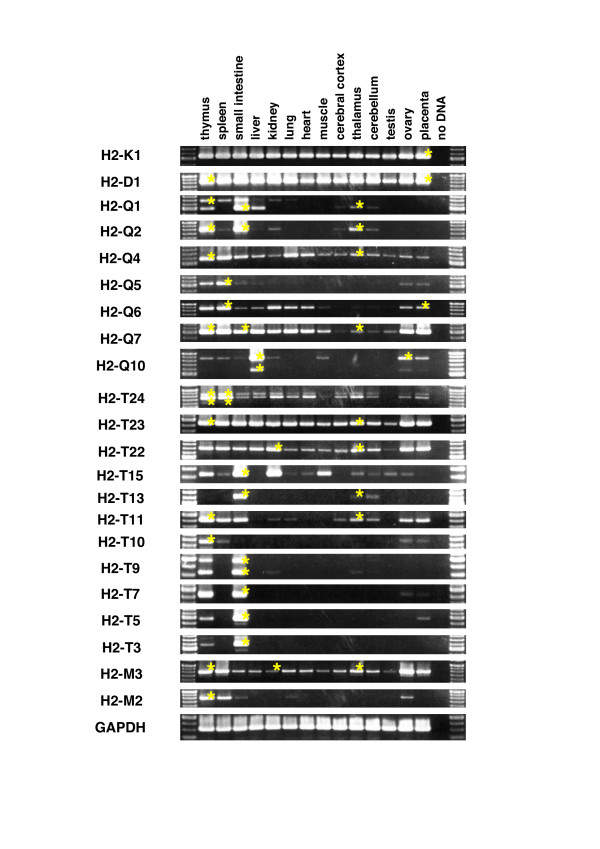
**Expression of *Mhc *class Ib genes in adult tissues**. RT-PCR was performed on total RNA isolated from tissues of C57BL/6J mouse. Identities of bands were confirmed by amplified sizes and by sequencing (indicated by yellow asterisk). The same reaction conditions were used for PCR.

To clarify the tissue expression patterns for each of the *Mhc *class Ib genes, we conducted RT-PCR analysis of the cDNAs isolated from various tissues of the adult mouse. Although it is difficult to analyze *Mhc *class I expression due to the sequence similarity of the *Mhc *genes (showing 60 – 95% identities in coding region; data not shown), we circumvented this disadvantage by designing the gene-specific primer sets that are listed in Table 1. Transcription of each *Mhc *class I gene was detected as shown in Figure 4. The gene identities of the amplified cDNAs were confirmed by direct sequencing of the RT-PCR-amplified fragments (indicated by yellow asterisks in Figure 4). Using the specific primer sets, we successfully amplify most of the identified *Mhc *class Ib genes, except for *H2-M5*, which may be expressed at very low levels and below the limit of detection of our RT-PCR assays. We obtained amplified fragments of the *H2-M5 *gene from the brain and thymus, but we were unable to detect amplified products in the other tissues (data not shown).

The gene expression patterns were classified into two types: tissue-wide or tissue-specific expression. *H2-Q4*, -*Q7*, -*T24*, -*T23*, -*T22 *and -*M3 *as well as the class Ia genes (*H2-K1 *and -*D1*) exhibited tissue-wide expression. In contrast, *H2-Q1*, -*Q2*, -*Q5*, -*Q6*, -*Q10*, -*T15*, -*T13*, -*T11*, -*T10*, -*T9*, -*T7*, -*T5*, -*T3 *and -*M2 *genes were expressed in a tissue-specific manner. Regardless of the tissue-wide or tissue-specific expression patterns, most of the class I genes were expressed in the thymus and intestine, both of which are critical organs for immune defense.

**Figure 5:**
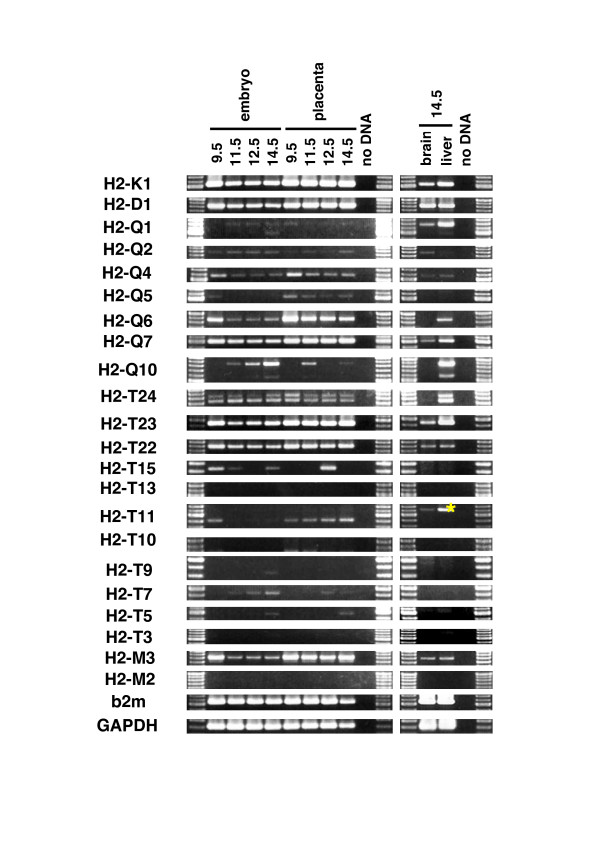
**Embryonic and placental expression of *Mhc *class Ib genes**. RT-PCR analysis was performed on total RNA isolated from E9.5–14.5 of C57BL/6J embryos and placentas. As for the E14.5 embryo, RT-PCR was conducted using cDNA as templates, derived from total RNA isolated from the brain and liver. Identities of bands were confirmed by amplified sizes and by sequencing (indicated by yellow asterisk). The same reaction conditions were used for PCR.

The tissue expression patterns of the genes *H2-T11 *and -*T10 *located within the duplicated D2 region (Figure 2) are more tissue-restricted than those of the respective paralogous genes *H2-T23 *and -*T22 *(Figures 4 and 5) that are located within the duplicated D1 region (Figure 2), confirming that major changes do occur in the expression profiles and functions of recently duplicated genes. Of particular note is the loss of expression in the liver, heart, muscle and testis by *H2-T11*, as previously reported for the liver [31], in comparison to its paralogous gene, *H2-T23*; and the loss of expression of *H2-T10 *in all tissues except the thymus, spleen, ovary and placenta in comparison to the tissue-wide expression by its paralogous gene *H2-T22*. The gene paralogs, *H2-T13*, -*T7 *and -*T5*, all showed tissue specific expression in the small intestine, except that the brains of adults also expressed the *H2-T13 *gene, the thymus and placenta expressed the *H2-T5 *gene and the thymus, ovary and placenta expressed *H2-T7 *(Figure 4).

The tissue expression patterns of the two flanking genes, *H2-T24 *and -*T3*, in the *H2-T *region are markedly different and may be among the oldest of the genes in this region. The centromeric *H2-T24 *gene was expressed widely, whereas the telomeric *H2-T3 *gene expression was restricted to the thymus and the small intestine (Figure 4) as previously reported [12,13].

As described above, the genes *H2-Q5*, -*Q6 *and -*Q7 *were probably generated by monogenic tandem duplications. In this regard, *H2-Q7 *showed the widest tissue expression, followed by *H2-Q6 *and then *H2-Q5*. This suggests that there might have been a gain or loss of tissue specificity with each gene duplication event. Of the other *H2-Q *genes, the most tissue-wide expression was by *H2-Q4*.

The *Mhc *gene expression in the brain is of particular interest because such genes could have a specific function in brain development and plasticity [17]. In this study, we identified 12 class Ib genes, *H2-Q1*, -*Q2*, -*Q4*, -*Q7*, -*T24*, -*T23*, -*T22*, -*T15*, -*T13*, -*T11*, -*M3 *and -*M5*, expressed in the brain. The *Mhc *gene expression in the brain warrants further investigation particularly to determine in what cells (neurons and/or various glial cells) and at what stages of brain development these genes are expressed.

### Expression of Mhc class Ib genes in embryos and placentas

Some *Mhc *genes are known to express and function during development in the embryo [32,33] and/or in the placenta [34]. Therefore, we determined which of the 20 class Ib genes were expressed in the embryo and placenta (Figure 4 and 5). The expression of some of the class Ib genes gradually increased (e.g. *H2-Q10 *and -*T7*) or decreased (e.g. *H2-Q6 *and -*M3*) during the course of development. The class Ib genes that were expressed widely in the adult tissues (*H2-Q4*, -*Q7*, -*T24*, -*T23*, -*T22 *and -*M3*) also tended to be expressed throughout the developmental stages. This observation suggests that the regions in which these class Ib genes are located may have an open or accessible chromatin configuration from the time of the first observable developmental stage. We could not detect *H2-T13*, -*T10*, -*T9*, -*T3 *and -*M2 *in the embryo or placenta, although *H2-T13 *(*Blastocyst MHC*) was previously shown to express in the placenta of B6 mice [34]. This negative result may be due to the developmental stage examined. Tajima et al. (2003) examined *Blastocyst MHC *gene expression at E3.5, E7.5 and E13.5 and expression at E13.5 was difficult to detect [34], while we analyzed gene expression at the developmental stages from E9.5 – E14.5.

We also examined the expression of class Ib genes in the brains of the E14.5 embryos (Figure 5). Nine genes (*H2-Q1*, -*Q2*, -*Q4*, -*Q7*, -*T24*, -*T23*, -*T22*, -*T11 *and -*M3*) were transcribed in the brains of the E14.5 embryos. All of them were also expressed in the adult brain (Figure 4), indicating that these gene products may have a functional role in both adult and embryonic brains.

From the RT-PCR analyses in Figure 4 and 5, we identified alternative splicing variants in the *H2-Q1*, -*Q10*, -*T24*, -*T11*, -*T9 *and -*M5 *(for *M5 *gene, see GenBank:AB378579) genes. The splicing patterns can be classified into four types: A) a common splicing pattern for class I gene, B) a loss of alpha2 domain, C) an unspliced second intron and D) an unspliced fourth intron. The type B variant was seen for *H2-Q10 *and -*M5 *expression, whereas type C was observed in *H2-Q1*, -*T11 *and -*T9 *expression. *H2-T24 *showed type D variant. It is of interest in future to determine whether these splicing variants have distinct or common functions. The type A and type B variants were previously reported for the *H2-T13 *(*Blastocyst MHC*) gene, and the RMA-S cell expressing the type B variant was protected from NK cell-mediated rejection via loading of its signal peptide onto the Qa-I molecules [34].

### Expression patterns between duplicated class Ib genes

Since local duplication in the *H2-T *region (Figure 2) have produced gene sets with high sequence similarity (Figures 1B,C and 2) even in the upstream promoter region (Figure 3), a redundant expression pattern was expected between the similar genes. However, as described above, the expression patterns between similar genes were mostly different. For example, *H2-T23 *was expressed widely, whereas the *H2-T11 *gene paralog showed a much more restricted expression pattern. This difference in expression between duplicated genes was especially remarkable for *H2-T22 *and -*T10 *expression (Figure 4) because the sequences of the upstream promoter regions of *H2-T22 *and -*T10 *are almost identical (Figure 3). In contrast, the *H2-T13*, -*T5 *and -*T7 *duplicated genes have similar nucleotide sequences, including within their promoter region, and similar expression patterns (predominantly in small intestine). This expression pattern, especially for *H2-T5 *and -*T7*, was almost the same as for *H2-T3 *that flanks these genes (Figure 2), but exhibited no similarity in the promoter sequence (Figure 3).

The co-expression of neighboring genes, such as *H2-T24 *to -*T22 *or *H2-T15 *to -*T3 *(Figure 2), may be regulated by 1) independent cis-acting regulatory elements for each gene that produce similar expression patterns, or by 2) a shared long-range regulatory element that operates over several genes (i.e. a long-range enhancer and/or a chromatin level regulation). Model 1 is appropriate for duplicated regions in which control regions are duplicated together with the coding sequence [35]. This is the most likely explanation for co-expression of *H2-T5 *and -*T7 *(Figure 3). The possibility that different promoter sequences produce a similar expression pattern might also be explained by model 1. The 2.8 kb promoter region of *H2-T3 *was shown previously to direct transgene expression in the epithelial cells of the small and large intestine [36]. Therefore, it will be of interest in future to examine whether the upstream regions of *H2-T5 *and -*T7 *have the same activity as that of *H2-T3 *(Figure 3). We think, however, it is unlikely that all the genes located between *H2-T24 *to -*T22 *or *H2-T15 *to *T3 *contain their own cis-regulatory element with similar function. Considering the order of the *H2-T *genes that show tissue-wide or tissue-specific expression, we rather favor model 2. The *H2-T *genes with tissue-wide expression are located within the same 40 kb centromeric portion of the *H2-T *region (*H2-T24 *to -*T22*), whereas the genes *H2-T15 *to -*T3 *located at the telomeric-end exhibited a tissue-specific expression pattern with most of them predominantly expressed in the small intestine (Figure 4). The region containing the genes from *H2-T15 *to -*T3 *with the restricted tissue expression spans as much as 150 kb, which is consistent with the possibility of a long-range regulation. The long-range regulation may provide a simple explanation of different expression patterns of similar genes (e.g. *H2-T22 *and -*T10*) and similar expression pattern of genes with distinct promoter regions (e.g. *H2-T5*, -*T7 *and -*T3*) over long distances. This model is supported by recent papers that reported that a special AT-rich binding protein 1 (SATB1), the most characterized matrix attachment regions (MARs)-binding protein (MBP), is involved in the tissue-specific chromatin organization of the human *MHC *class I locus and its expression profile [37,38].

The mouse is known to have strain-specific gene duplications in the *H2-T *region with a number of duplicated *H2-T *gene differences between strains producing considerable variability between haplotypes [39,40]. The genomic features, organization and the expression patterns of the *H2-T *genes in other mouse strains warrant a comparative analysis. The expression pattern analysis of rat *Mhc *class Ib genes [41] may also provide clues for our hypothesis for the long-range regulation of duplicated class Ib gene expression. In addition, an investigation of gene duplications in genetically modified mice may help to distinguish between the different models involved in the regulation of duplicated gene expression. We are currently generating chromosomally engineered mice towards these ends.

## Conclusion

We have identified 21 transcribed *Mhc *class Ib genes in the *H2-Q*, -*T *and -*M *regions and examined their expression patterns within a wide array of developmental and adult mouse tissues. Some of the class Ib gene products were expressed tissue-wide, while others were expressed in a tissue-restricted manner. These results provide a basis to select important candidate *Mhc *class Ib genes for future functional validation studies. For example, we found 12 brain-expressed class Ib genes that could have neuronal and other functions in brain development and plasticity. We also found that genes expressed tissue-wide are located in the centromeric region, whereas the tissue-specifically expressed genes are located towards the telomeric end of the *H2-T *region where the number of genes has been increased by local duplication. In this region, there are genes that showed distinct expression patterns in spite of their similar nucleotide sequences, and there is a gene pair that has a similar expression pattern, but dissimilar promoter sequence regions. From these results, the presence of a long-range regulation of *H2-T *genes is suggested, although we cannot dismiss the possibility that nucleotide changes in the promoter and enhancer regions have contributed to the loss or gain of tissue-wide expression. Since this region has diversified not only between rodent species, but also between mouse strains, it should be a good model region to address the relationship between genomic organization and expression patterns.

## Methods

### Sequence analysis

The genomic sequences of the *H2 *region used in this study were obtained from the public databases at the NCBI Entrez Genome Project ID 9559 [42] and the Ensembl Mouse Genome Project [43]. Although we first analyzed the NT_039650.2 genomic contig by using a GENSCAN program [44] to identify the *Mhc *class Ib genes, we finally utilized the NCBI Mouse Build 36 containing the nearly completely annotated sequence of this region, which was released on June 20^th^, 2006. Dot matrix analysis was performed on these genomic sequences to detect duplicated regions by using Harrplot Ver. 2.0 as part of the computer software GENETYX package. Complete or partial coding sequences of each *Mhc *class I gene was first predicted by GENSCAN, referred to the annotation, and finally confirmed by the sequencing of RT-PCR products. These coding sequences (nucleic acids) were aligned by the ClustalW program version 1.83 at DDBJ [45] using the default setting and Kimura's two-parameter method to estimate the evolutionary distances. The final outputs as radial phylogenetic trees were generated with the TreeView drawing software. The sequences used for the phylogenetic tree analyses are listed in Table 2 (shown in "Ensembl transcript ID" column for *H2-Q1*, -*Q2*, -*Q5*, -*Q6*, -*Q7*, -*Q10*, -*T24*, -*T13*, -*T11*, -*T10*, -*T7 *and -*T3*, in "NCBI accession" column for *H2-Q4*, and in the "Determined in this study" column for *H2-T23*, -*T22*, -*T15*, -*T9*, -*T5*). PipMaker analyses were performed on selected *Mhc *class Ib gene sequences to visualize the DNA sequence similarities [46]. The genomic sequences analyzed by PipMaker contained the regulatory region 3 kb upstream from ATG start codon and the untranslated downstream region 1 kb from the stop codon in addition to the exon and intron sequences.

### Reverse transcriptase-polymerase chain reaction (RT-PCR)

The mRNA expression of *Mhc *class Ib genes was determined by RT-PCR analysis. Total cellular RNA was isolated from the thymus, spleen, small intestine, liver, kidney, lung, heart, skeletal muscle, cerebral cortex, thalamus, cerebellum, testis and ovary of adult C57BL/6J mice, and the embryo (E9.5 – E14.5, where embryonic day 0.5 [E0.5] was defined as midday (noon) of day 1 when a vaginal plug was detected after overnight mating.), placenta (E9.5 – E14.5), and embryonic (E14.5) brains and livers of C57BL/6J mice using the guanidine isothiocyanate/CsCl ultracentrifugation method. Complementary DNA (cDNA) was synthesized from isolated RNA using the Gene Amp RNA-PCR core kit (Applied Biosystem) with the oligo-dT primer and 2 μg RNA as template in a 40 μl volume according to the manufacture's protocol. An aliquot of 0.5 μl from the 40 μl of the cDNA was used for RT-PCR reactions of all cDNA samples. The PCR was performed in 20 μl of a total reaction volume under the following conditions: cDNA was denatured at 95°C for 5 min, followed by 35 cycles of amplification (95°C for 45 s, 58°C for 30 s and 72°C for 1 min) and 5 min at 72°C. The PCR primers used for the amplifications are listed in Table 1 (see also additional file 1). The primer sets were manually designed to amplify specific *Mhc *class Ib genes by locating the gene specific polymorphisms within 5-bp of the 3' end as much as possible. All primers were designed within putative cDNA to flank or cross at least one exon-intron border. Resultant RT-PCR products were directly sequenced to verify their identity.

### 3' Rapid amplification of cDNA end (RACE) and cloning of class Ib cDNAs

To determine the complete cDNA sequences of *H2-T5*, -*T15*, -*T22*, -*T23 *and -*M5*, 3'RACE was performed using thymus or duodenum RNAs as template, the oligo-dT-primer with adapter (GGCCACGCGTCGACTAGTACT_17_.), and the forward primers listed in Table 1. The 3'RACE products were cloned into pBSII plasmid (STRATAGENE). RT-PCR covering the translation start site was done using the following forward primers designed from the genomic sequences around the translation start codon (ATG) as predicted by GENSCAN program:

*H2-T5*; TCTCCTGTATCATCATTCCCAGAT,

*H2-T15*; ACTGTACTGAGCTCTCTCTATCCCA,

*H2-T22*; AGTTTATAAAGCTGTCCAAGATCT,

*H2-T23*; GATTCAGGTTCCTCACAGACCCAG,

*H2-M5*; TGTATGAGAAGCCCTGCGCTCT, and the reverse primer listed in Table 1. The products were also cloned into pBSII plasmid. The nucleotide sequences of the 3'RACE and RT-PCR products were combined and analyzed.

## List of abbreviations

Mhc: major histocompatibility complex; RT-PCR: Reverse transcriptase-polymerase chain reaction.

## Authors' contributions

MO designed and performed the experiments, conducted genome analyses, prepared the manuscript, and is responsible for this study. HI is the director of the laboratory and gave suggestions for this study. JKK helped in editing the manuscript and in interpreting the genome analysis. SY carried out the preparation, cloning and sequencing of cDNA, and participated in the design of the study. All authors have read and approved the final manuscript.

## Supplementary Material

Additional file 1 **Primer positions**. Positions of primers were indicated in alignment of *Mhc *class I sequences. Forward and reverse primers were shown in red and blue, respectively. Click here for file
