# Supplementary material for: Major histocompatibility complex (Mhc) class Ib gene duplications, organization and expression patterns in mouse strain C57BL/6
Source: BMC Genomics. 2008 Apr 17;9:178. doi: 10.1186/1471-2164-9-178 (PMC2375909; doi:10.1186/1471-2164-9-178)
Supplement: Additional file 1 — Primer positions. Positions of primers were indicated in alignment of Mhc class I sequences. Forward and reverse primers were shown in red and blue, respectively. [file 1471-2164-9-178-S1.doc]

**Primer positions**

Red: forward primers

Blue: reverse primers

K1 ------------------------------------------------------------

D1 ------------------------------------------------------------

Q1 ------------------------------------------------------------

Q2 ------------------------------------------------------------

T23 ------------------------------------------------------------

T11 ------------------------------------------------------------

T22 --------------------------------------------ATGTCCTGGGTCCTCA

T10 --------------------------------------------ATGTCCTGGGTCCTCA

M5 --------------------------------------------------------ATGA

T15 --------------------------------------------------------ATGG

T9 --------------------------------------------------------ATGG

M3 --------------------------------------------------------ATGG

M2 --------------------------------------------------------ATGG

T3 --------------------------------------------------ATGAGGATGG

T13 ------------------------------------------------------------

T7 ------------------------------------------------------------

T5 ------------------------------------------------------------

Q5 ------------------------------------------------------------

Q7 ------------------------------------------------------------

Q6 ------------------------------------------------------------

Q4 ATGGCGTCAACAATGCTGCTTCTGCTGGTGGCAGTCGCCCAGACCCTGATCGAGATCCGC

Q10 --------------------------------------------------------ATGG

T24 ------------------------------------------------------------

K1 -----ATGGTACCGTGCACGCTGCTC--------CTGCTGTTGGCGGCCG---CCCTGGC

D1 -----ATGGCTCCGCGCACGCTGCTC--------CTGCTGCTGGCGGCCG---CCCTGGC

Q1 -----ATGGCACTGGGAAGGCTGCTC--------CTGCTGCTGGCAGCCG---CCCTGAC

Q2 -----ATGGCGCTGCGAAGGCTGCTC--------CTGCTGTTGGTGGCCG---CCCTGAA

T23 -----ATGTTGCTTTTTGCCCACTTG--------CTTCAGCTGCTGGTCAGCGCCACAGT

T11 -----ATGTTGCTTTTTGCCCACTTG--------CTCCAGCTGCTGGTCAGCGCCACAGT

T22 GGGCCGCTGTGGTCTGCGCCCTTCTC--------CTGCAGCTGGATGCCAGACCAT---C

T10 GGGCTGCTGTGGTCTGCGCCCTTCTC--------CTGCAGCTGGATGCCAGACCAT---C

M5 GAAGCCCTGCGCTCTCTACCCTCCTA--------TCCTTGCTGCTCACCGGAGCTTTGGC

T15 GGACCCTTACGAGTTGCCCTCTACTT--------CTCCTGCTTCTGGCGACCGCCCTGGC

T9 GGACCCTTACGAGTTGCCCTCTACTT--------CTCCTGCTGCTGGCGACCGCCCTGGC

M3 GGTCCTCAAGCAACCGCGCTCTCCTG-----CACATGGTGGTGGTCTCTTTGGCTGTTAC

M2 AGACTTCAGCATTCTTCACTCTCCTCT---TCACTCTCTACCTGCTGCTTGGGCCCTTGC

T3 GGACCATGGTGCCTGGCACCCTCCTG--------ATCCTCCTGGCTGCCT---CACAAGG

T13 -----ATGGCGCAGCGAACGCTGTTC--------CTGCTGCTGGCGGCCG---CCCTGAC

T7 -----ATGGCGCAGCGAATGCTGCTC--------CTGCTGCTGGCGGCCG---CCCTGAC

T5 -----ATGGCACCGCGAACGCTGCTC--------CTGCTGCTGGCCGCTG---CCCTGAT

Q5 -----ATGGCTCTAACAATGCTGCTC--------TTGCTGGTGGCGGCCG---CCCTGAC

Q7 -----ATGGCTCTAACAATGCTGCTC--------TTGCTGGTGGCGGCCG---CCCTGAC

Q6 -----ATGGCTCTAACAACGCTGCTC--------TTGCTGGTGGCGGCCG---CCCTGAC

Q4 GCGGCCCCGCGCCCTGCTCCCCTCCCGGCCCGCTCACCCGCCGGGGGTCCCGGAAGGAGT

Q10 GGGCGATGGCGCCGCGCACGCTGCTC--------CTGCTGCTGGCGGCTG---CCCTGGC

T24 -----------ATGTGGGCTCTTATC--------TTCTGGCTTCTGAGTCACCCCCAGGA

*

K1 TCCGACTCAGACC------------CGCGCGGGCCCACACTCGCTGAGGTATTTCGTCAC

D1 CCCGACTCAGACC------------CGCGCGGGCCCACACTCGATGCGGTATTTCGAGAC

Q1 CCTGACCAAAACC------------GGAGCGGGCTCACACTCGCTGCGGTATTTCGAGAC

Q2 ACTGACTGAGACA------------CGCGCGGGCTCACACTCGCTGCGGTATTTCACCAC

T23 CCCGACCCAGAGT------------------AGCCCACACTCGCTGCGGTATTTCACCAC

T11 CCCGACCCAGAGT------------------AGCTCACACTCGCTGCGGTATTTCCACAC

T22 CTGGACTCGGATC------------CCTTTGGG---------------GTATTTCTACAC

T10 CTGGACTCGGATC------------CCTTTGGGTTCACACTCGCTTAGGTATTTCTACAC

M5 CTTGACCCTGGTT------------CGCGCAGGCATCCATTCCTTGCAGTTTTTTGCCAC

T15 CCCGACCCAAGCG------------GGCAAGAGCTCACACTCGCTGCGGTATTTCGCCAC

T9 CCCGACCCGAGCG------------GGCAAGAGCTCACACTCGCTGCGGTATTTCGCCAC

M3 CCAAACA------------------GGTTCTGGCTCACATTCACTGCGCTATTTCCACAC

M2 CCTGGCACAGACCTTTAAAGG--TGAGTGCAGGATCCCACTCCTTGCGGTACTTCGACAT

T3 CCAGACCCAGACC------------TGCCCAGGCTCACACTCGCTGAGGTACTTCTACAC

T13 TATGATCGAGACTCGCGCGG------------GCCCACACTCGATGCGATATTTCGAGAC

T7 CCTGATCGAGACCCGCGCGG------------GCTCACACTCGATGCGGTATTTCGAGAC

T5 ACTGACGGAGACACGCGCGGTGTCCCGGCCCGGCCCACACTCGATGCGGTATTTCGAGAC

Q5 CCTGATCGAGACC------------CGCGCGGGCCCACACTCGCTGCGGTATTTCCACAC

Q7 CCTGATCGAGACC------------CGCGCGGGCCAACACTCGCTGCAATATTTCCACAC

Q6 CCTGATCGAGACC------------CGCGCGGGCCCACACTCGCTGCGGTATTTCCACAC

Q4 TCGGGGTCTCACCGCGCCCTG----CCTCCAGGCCCACACTTGCTGAGTTATTTCTACAC

Q10 CCCGACCCAGACC------------CAGGCAGGCTCACACTCCATGAGGTATTTCGAAAC

T24 C--GGCGGAGCCC-------------------GGTCGCACTCTCTGCATTACTGTTACTC

* * *

K1 CGCCGTGTCCCGGCCCGGCCTCGGGGAGCCCCGGTACATGGAAGTCGGCTACGTGGACGA

D1 CGCCGTGTCCCGGCCCGGCCTCGAGGAGCCCCGGTACATCTCTGTCGGCTATGTGGACAA

Q1 CTCGGTGTCCCGGCCCGGCTTCGGGAAGCCCCGGTTCATCTCTGTCGGCTACGTGGACGA

Q2 CGCCGTGTCCCGGCCTGGCCTCGGGGAGCCCCGGTTCATTATCGTCGGCTACGTGGACGA

T23 CGCCGTGTCCCGGCCCGGCCTCGGGGAGCCCCGGTTCATCATTGTCGGCTACGTGGACGA

T11 CGTCGTATCCCGGCCCGGCCTCGGAGAGCCCCGGTTCATCATTGTCGGCTACGTGGACGA

T22 CGCTGTGTCCCGGCCTGGCCTTGGGGAGCCCTGGTTCATAATCGTCGGCTATGTGGACGA

T10 CGCTGTGTCCCGGCCTGGCCTTGGGGAGCCCTGGTTCATAATTGTCGGCTATGTGGACGA

M5 CACCATGACCCAGCCTGGTTTGAGGGAGCATTCCTTCATCTTTGTCGTCTTCGTGGACGA

T15 TGCCATATCCAGGCCAGGCTTCGGGGAGCCTCGGTTCACCGCCGTGGGTTACGTGGACGA

T9 TGCCATGTCCAGGCCAGGCTTAGGGGAGCCCCGGTTCACCGCCGTGGGTTACGTGGACGA

M3 TGCGGTGTCACGGCCGGGCCGTGGGGAGCCCCAGTATATCTCTGTGGGCTATGTTGACGA

M2 CGCAGTGTCAAGACCTGGCCTAGAGGAGACCCACTACATGACTGTTGGCTATGTGGATGA

T3 CGCCTTGTCCCGACCTGCAATCAGCGAACCGTGGTACATAGCTGTGGGCTACCTGGATGA

T13 CGCAGTGTTCCGGCCCGGCCTCGGGGAGCCCCGGTTCATCTCTGTCGGCTACGTGGACAA

T7 CGCCTTGTCCCGCCCTGGCCTCCGGGAACCCCGATTCATCTCTGTCGGCTACGTGGACGA

T5 CGCCGTGTCCCGGCCCGGCCTCGGGGAGCCCTGGTTCATCTCTGTCGGCTACGTGGACGA

Q5 CGCTGTGTCCCGGCCCGGACTCGGGGAGCCCCGGTTCATCATCGTCGGCTACGTGGACGA

Q7 CGCTGTGTCCCGGCCCGGCCTCGGGGAGCCCTGGTTCATCTCTGTCGGCTACGTGGACGA

Q6 TGCTGTGTCCTGGCCCGGCCTCGTGGAGCCCCGGTTCATTATCGTCGGCTACGTGGACAA

Q4 CTCCGTGTCCCGGCCGGGCCTTGGGGAGCCCCGGTTCATCTCTGTCGGTTACGTGGACAA

Q10 CTCCGTTTCCCGGCCGGGCCTTGGGGAGCCCCGGTTCATTATTGTCGGTTACGTGGACGA

T24 AGCTGTGACTGAGCCGGGCCCGGGAGTTCCTTCATTTTTCGCCAGTGGCTTCTTAGATAA

* ** * * * * * ** *

K1 CACGGAGTTCGTGCGCTTCGACAGCGACGCGGAGAATCCGAGATATGAGCCGCGGGCGCG

D1 CAAGGAGTTCGTGCGCTTCGACAGCGACGCGGAGAATCCGAGATATGAGCCGCGGGCGCC

Q1 CACGCAGTTTGTGCGCTTCGACAGCGACGCGAAGAATCCGAGATATGAGCCGCGGGCGCC

Q2 CACGCAGTTCGTGCGCTTCGACAGCGACGCGGAGAATCCGAGGATGGAGCCGCGGGCGCC

T23 CACGCAGTTCGTGCGCTTCGACAGCGACGCGGAAAATCCGAGGATGGAGCCTCGGGCGCG

T11 CACGCAGTTCGTGCGCTTCGACAGCGACTCGGAGAATCCGAGGATGGAGCCTCGGGCGCG

T22 CATGCAGGTCCTGCGCTTCAGCAGCAAGGAGGAGACTCCGAGGATGGCACCC--------

T10 CATGCAGGTCCTGCGCTTCAGCAGCAAGGAGGAGACTCCGAGGATGGCACCC--------

M5 CACACAGTTCCTGTGCTACAACAATAAGGGGAAAAATCAGAGAATGGAGCCACGCGCTCT

T15 CACACAGTTCATGCGCTTCGACAGCGACTCTGAGAATCCCAGGGCCGAGCCATGCAAGCC

T9 CACACAGTTCATGCGCTTCGACAGCGACTCTGAGAATCCCAGGGCCGAGCCATGCAAGCC

M3 CGTGCAGTTTCAGCGCTGTGATAGCATTGAGGAAATTCCGAGGATGGAACCTCGTGCACC

M2 CACAGAGTTTGTGCATTTTGACAATGAGGCTGAGAATCCGAGGTTTGAGCCCCGAGTGCC

T3 CACTCAGTTCGTGCGCTTCAACAGCTCAGGGGAGACTGCGACATATAAGCTAAGTGCGCC

T13 CACGCAGTTCGTGAGCTTCGACAGCGATGCGGAGAATCCGAGATCTGAGCCGCGGGCGCC

T7 CACGCAGTTCGTGCGCTTCGACGGCGACGCGGAGAATCCGAGGTATGAGCCGCGGGCTCC

T5 CACGCAGTTCGTGCGCTTCGACGGCGACGCGGAGAATCCGAGGATGGAGCCGCGGGCGCC

Q5 CACGCAGTTCGTGCGCTTCGACAGCGACGCGGAAAATCCGAGGATGGAGCCGCGGGCGCG

Q7 CACGCAGTTCGTGCGCTTCGACAGCGATGCGGAAAATCCGAGGATGGAGCCGCGGGCGCG

Q6 CAAGCAGTTCGTGCGCTTCGACAGCGACGCGGAAAATCCGAGGATGGAGCCGCGGGCGCG

Q4 CACGGAGTTCGTGCGCTTCGACAGCGACGCGGAGAATCCGAGATATGAGCCGCGGGCACC

Q10 CACGCAGTTCGTGCGCTTCGACAGCGACGCGGAGACTCCGAGGATGGAGCCGCGGGCGCC

T24 CCAGCCCTTCATCCACTACGACAGCA----GGAGCAT--GAAGGCAGAGCCTTGTGCTGA

* * * * * * *

K1 GTGGATGGAGCAGGAGGGGCCCGAGTATTGGGAGCGGGAGACACAGAAAGCCAAGGGCAA

D1 GTGGATGGAGCAGGAGGGGCCGGAGTATTGGGAGCGGGAAACACAGAAAGCCAAGGGCCA

Q1 GTGGATGGAGCAGGAGGGGCCGGAGTATTGGGAACGGAACACACGGAGAGTCAAGGGCAG

Q2 GTGGATGGAGCAGGAGGGGCCGGAGTATTGGGAGCGGAACACACAGGTCTCCAAGGAAAA

T23 GTGGATTGAGCAGGAGGGGCCGGAGTATTGGGAGCGGGAGACTTGGAAAGCCAGGGACAT

T11 GTGGATTGAGCAGGAGGGGCCGGAGTATTGGGAGCGGGAGACTCGGAAAGCCAGGGACAT

T22 -TGGCTGGAGCAGGAGGAAGCAGATAACTGGGAGCAGCAGACTCGTATAGTCACAATTCA

T10 -TGGCTGGAGCAGGAGGAAGCAGATGACTGGGAACAGCAGACTCGTATAGTCACAATTCA

M5 GTGGGTGAAGCAGATGGGGCCAGAGTATTGGGAACAGCAGACCAGGACTGTCAAGGTCAT

T15 ATGGGTGGAACAGATGGAGCCCGAATATTGGGAGCAGGAGACGCGGAAATTCAAGGAGCA

T9 ATGGGTGGAACAGATGGAGCCCGAATATTGGGAGCAGGAGACGCGGAAATTCAAGGAGCA

M3 TTGGATGGAGAAGGAAAGACCAGAGTACTGGAAGGAGCTGAAACTCAAGGTCAAGAATAT

M2 TTGGATGGAACAGATGGGACAGAAGTACTGGGATGACCAGACACGCATTGCGAAAGCTGC

T3 ATGGGTGGAGCAAGAGGGGCCCGAGTATTGGGCGAGAGAGACAGAGATCGTCACAAGCAA

T13 GTGGATGGAACAGGAGGGACCGGAGTATTGGGAGCGGGAAACACAGATCGCCAAGGACAA

T7 ATGGATGGAGCACGAGGGGCGGGAGTATTGGGAGCGGGAGACACAGATCGCCAAGCGCAA

T5 CTGGATGGAGCAGGAGGGGCCGGAGTATTGGGAGCGGGAGACACAGATCGCCAAGGGCCA

Q5 GTGGATGGAGCAGGAGGGGCCGGAGTATTGGGAGCGGGAGACACAGGTCGCCAAGGGCCA

Q7 GTGGATGGAGCAGGAGGGGCCGGAGTATTGGGAGCGGGAGACACAGATCGCCAAGGGCCA

Q6 GTGGATGGAGCAGGAGGGGCCGGAGTATTGGGAGCGGGAGACACAGAAAGCCAAGGGCCA

Q4 GTGGATGGAGCAGGAGGGGCCGGAGTATTGGGAGCGGGAAACACAGAAAGCCAAGGGCAA

Q10 TTGGATGGAGCAGGAGGGGCCGGAGTATTGGGAGCGGGAGACACAGAGAGCCAAGGGCAA

T24 CTGGCTGAGGGAAAATGCAC---AGTACTTCACTCATGAGACTGAGGTTTTCACCAATCG

*** * * * * * * *

K1 TGAGCAGAGTTTCCGAGTGGACCTGAGGACCCTGCTCGGCTACTACAAC-----------

D1 AGAGCAGTGGTTCCGAGTGAGCCTGAGGAACCTGCTCGGCTACTACAAC-----------

Q1 TGAGAAGAGATTCCAAGAGAGCCTGAGCACCCTGCTCAGCTACTACAAC-----------

Q2 TGAGCAGAGTTTCCGAGTGAGCCTGGGGACCGCACTGAGCTACTACAAC-----------

T23 GGGGAGGAACTTCAGAGTAAACCTGAGGACCCTGCTCGGCTACTACAAT-----------

T11 GGGGAGGAACTTCAGAGTAAACCTGAGGACCCTGCTCGGCTACTACAAT-----------

T22 AGGACAGCTGTCTGAAAGGAATCTGATGACCCTGGTTCATTTTTACAAC-----------

T10 AGGACAGCTGTCTGAAAGGAATCTGATGACCCTGGTTCATTTTTACAAC-----------

M5 TGAGAAGATTGCCCTAGTGAATTTGCAGGAGGCCATGGACATCTACAAC-----------

T15 CACCCAGAATTTCCGAACTTGCCTTTACAACCTACTCCACCTGTATAAC-----------

T9 CACCCAGAATTTCCGAACTTGCCTTTACAACCTACTCCACCTGTACAAC-----------

M3 TGCACAAAGTGCCAGAGCAAACCTTCGGACCCTGCTCCGCTACTACAAC-----------

M2 AGAACAGCAGATTAGAGTGTACTTTCAGAAACTGCGAGACTACTACAAC-----------

T3 TGCACAGTTTTTCCGCGAGAATCTGCAGACTATGCTGGACTACTACAAC-----------

T13 TGAGCAGAGTTTCGGATGGAGCCTGAGGAACCTGATCCACTACTACAAC-----------

T7 TGAGCAGAGTTTCCGAGGGAGCCTGAGGACCGCGCAGCGCTACTACAAC-----------

T5 TGAGCAGGGTTTCCAAGGGAGCCTGAGGAACCTGCTCCACTACTACAAC-----------

Q5 TGAGCAGAGTTTCCAAGGGAGCCTGAGGACTGCACAGAGCTACTACAAC-----------

Q7 TGAGCAGAGTTTCCGAGGGAGCCTGAGGACCGCACAGAGCTACTACAAC-----------

Q6 TGAGGAGAGTTTCCGAGTGAGCCTGAGGACCGCACAGAGATACTACAAC-----------

Q4 TGAGCAGATTTTCCGAGTGAACCTGAGGACCCTGCTCAGCTACTACAAC-----------

Q10 TGAGCAGAGTTTCCATGTGAGCCTGAGGACCCTGCTCGGCTACTACAAC-----------

T24 GATGAAGATTTTCCAGTTGAGCCTGAGAAACATTCGGCAATACTACAACAGCTCTGGTAC

* ** **

K1 -------CAGAGCAAGGGCGGCT------------------CTCACACTATTCAGGTGAT

D1 -------CAGAGCGCGGGCGGCT------------------CTCACACACTCCAGCAGAT

Q1 -------CAGAGCAAGGGCGGCA------------------TTCACACCTTCCAGAAGTT

Q2 -------CAGAGCAAGGGAGGCT------------------CTCACACTCTCCAGTGGTT

T23 -------CAGAGTAACGACGAAT------------------CTCACACGCTGCAGTGGAT

T11 -------CAGAGTAAGGACGAAT------------------CTCACACGCTGCAGTGGAT

T22 -------AAGAGCATGGACGACT------------------CTCACACACTACAGTGGCT

T10 -------AAGAGCATGGACGACT------------------CTCACACACTACAGTGGCT

M5 -------CACAGCAAGGATGGCT------------------CTCACGTCTTTCAGTGTGT

T15 -------CAGAGTCAGGACGGTC------------------CCCACACAATCCAGGATAT

T9 -------CAGAGTCAGGACGGTC------------------CCCACACTATCCAGGATAT

M3 -------CAGAGTGAAGGCGGCT------------------CTCACATCCTGCAGTGGAT

M2 -------CAGAGCCAGAACAGTT------------------CTCACACCATCCAAAGGAT

T3 -------CTGAGTCAAAATGGCT------------------CTCACACCATCCAGGTGAT

T13 -------CAGAGCAAGGGCGGCT------------------TTCACACATTCCAGCGGTT

T7 -------CAGAGTGAAGGCGGCC------------------TTCACACGTTCCAGCTGTT

T5 -------CAGAGCGCGGGCGGCT------------------CTCACACATTTCAGAGGAT

Q5 -------CAGAGCAAGGGCGGCT------------------CTCACACACTCCAGTGGAT

Q7 -------CAGAGCAAGGGCGGCT------------------CTCACACACTCCAGTGGAT

Q6 -------CAGAGCAAGGGCGGCT------------------CTCACACACTCCAGTGGAT

Q4 -------CAGAGCGCGGGCGGCT------------------CTCACACTATTCAGGTGAT

Q10 -------CAGAGCGAGAGCGGCT------------------CTCACACGATCCAGTGGAT

T24 CCAGAGTCAGAGAGCAGATGGCTTCCGCCAGCAAGCAGGCCCTCACACCCTCCAGTTCAC

** *** * **

K1 CTCTGGCTGTGAAGTGGGGTCCGACGGGCGACTCCTCCGCGGGTACCAGCAGTACGCCTA

D1 GTCTGGCTGTGACTTGGGGTCGGACTGGCGCCTCCTCCGCGGGTACCTGCAGTTCGCCTA

Q1 GTCTGGCTGTGATCTGGGGTCAGATGGGCGCCTTCAAAGCGGGTACCTGCAGTTCGCCTA

Q2 GGTTGGCTGTGACCTGGGGCCAGACGGAAGCCTACTCCGAGGGTATGAGCAGTCTGCCTA

T23 GTACGGCTGCGACGTGGGGCCCGATGGGCGCCTGCTCCGCGGGTATTGTCAGGAGGCCTA

T11 GTACGGCTGCGACGTGGGGCCCGATGGGCGCCTGCTCCGCGGGTATTGTCAGGAGGCCTA

T22 GCAAGGCTGCGATGTGGAGCCAGATCGGCACCTGTGTCTCTGGTACAACCAGCTCGCCTA

T10 GCAGGGCTGCGATGTGGAGCCAGATCGGCACCTGTGTCTCTGGTACAACCAGCTCGCCTA

M5 GTATGGCTGCGAAGTGGGGCCAGATGGACTCTTCCTCCGTGGGCATGAGAAGCACGCATA

T15 GCACGGATGCTACGTGGGACCAGATGGTCAGTTCCTACACGGGCACTATCAACATGCCTA

T9 GCACGGATGCTACGTGGGACCAGATGGTCAGTTCCTACACGGGCACTATCAACATGCCTA

M3 GGTTTCCTGTGAGGTAGGGCCAGACATGCGCCTTCTTGGAGCCCACTATCAGGCTGCGTA

M2 GACTGGCTGCTACATTGGACCAGACGGGCACCTACTTCACGCATACCGTCAGTTTGGTTA

T3 GTATGGCTGTGAGGTGGAGTTCTTCGGGAGCCTCTTCCGCGCTTATGAGCAGCATGGCTA

T13 GTCTGGCTGTGACATGGGGTTGGACGGGCGCCTCCTCCGCGGGTACCTGCAGTTCGCTTA

T7 GTTCGGCTGTGACGTGGGGTCGGACGGGCGCCTCCTCCGCGGATACCTACAGTTCGCTTA

T5 GTACGGCTGTGACCTGGGGTCGGACGGGCGCCTCCTCCGCGGGTACTGGCAGTTCGCTTA

Q5 GTATGGCTGTGACATGGGGTCCGACGGGCGCCTCCTCCGCGGGTACCTGCAGTTCGCCTA

Q7 GTATGGCTGTGACATGGGGTCCGACGGGCGCCTCCTCCGCGGGTACCTGCAGTTCGCCTA

Q6 GTATGGCTGTGACGTGGGGTCCGACGAGCGCCTCCTCCGCGGGTACCTGCAGTTCGCCTA

Q4 CTCTGGCTGTGAAGTGGGGTCCGACGGGCGCCTCCTCCGCGGGTACCAGCAGTTCGCCTA

Q10 GTATGGCTGTAAAGTGGGGTCCGACGGGCGCTTCCTCCGCGGGTACCTGCAATACGCATA

T24 ATACGGCTGTGAGATGCGGTACAACAGGACCACA------GGGCATTGGCAGTATGGCTA

** * * * * * **

K1 CGACGGCTGCGATTACATCGCCCTGAACGAAGACCTGAAAACGTGGACGGCGGCGGACAT

D1 TGAAGGCCGCGATTACATCGCCCTGAACGAAGACCTGAAAACGTGGACGGCGGCGGACAT

Q1 TGATGGCCTTGATTACATCGCCCTGAATGAAGACCTGGAAACCTGGACAGCAGCAGATGT

Q2 CGATGGCCGCGATTACCTCGCCCTGAATGAGGATCTGATAACGTGGACAGCGGCGGACCT

T23 CGATGGCCAGGATTACATCTCCCTGAACGAGGACCTGCGTTCCTGGACCGCGAATGACAT

T11 TGATGGCCAGGATTACATCTCCCTGAACGAAGACCTGCGCTCCTGGACCGCGACCAACTT

T22 TGATAGCGAGGATCTCCCCACCCTGAACGAAAACCCAAGTTCCTGTACAGTGGGAAACAG

T10 TGATAGCGAGGATCTCCCCACCCTGAGCGAAAACCCAAGTTCCTGTACAGTGGGAAACAG

M5 CGACGGCCGCGATTACCTCACCCTAAGCCCGGACCTGCACTCCTGGGTCGCAGGCGACAC

T15 TGATGGCCACGATTACATCACCCTGAACGAGGACCTGAGCTCCTGGACCGCAGCAGATGC

T9 TGATGGCCACGATTACATCACCCTGAACGAGGACCTGAGCTCCTGGACCGCAGCAGATGC

M3 TGATGGCTCAGATTACATCACCCTGAATGAAGACCTGAGCTCCTGGACTGCAGTGGACAT

M2 TGATGGGCAAGATTACCTCACTCTGAATGAAGATCTGAGCACCTGGACTGCAGCAGATGC

T3 TGATGGCCGCGATTACATTGCCCTCAATGAAGATCTGAAAACGTGGACAGCAGCAGACAC

T13 TGATGGCCGCGATTACATCACCCTGAACGAAGACCTGAAAACGTGGATGGCGGCGGATCT

T7 TGATGGCCGAGATTACATCGCCCTGAACGAAGACTTGAACACGTGGACGGCGGCGGACCT

T5 CGACGGCAGCGATTACATCGCCCTGAACCAAGACCTGAAAACGTGGACGGCGGCGGACTT

Q5 TGAAGGCCGCGATTACATCGCCCTGAACGAAGACCTGAAAACGTGGACGGCGGCGGACAT

Q7 TGAAGGCCGCGATTACATCGCCCTGAACGAAGACCTGAAAACGTGGACGGCGGTGGACAT

Q6 TGAAGGCCGCGATTACATCGCCCTGAACGAAGACCTGAAAACCTGGACGGCGGCGGACAT

Q4 CGACGGCCGCGATTACATCGCCCTGAACGAAGACCTGAAAACGTGGACGGCGGCGGACAT

Q10 CGACGGCCGCGATTACATTGCCCTGAACGAAGACCTGAAAACGTGGACGGCAGCGGACGT

T24 CGATGGCAGTGATTACCTGACCTTGGACTTGGGCTCTATGCAATATATAGCAGCCACCTT

** * *** * * * * *

K1 GGCGGCGCTGATCACCAAACACAAGTGGGAGCAGGCTG-GTGAAG--CAGAGAGACTCAG

D1 GGCGGCGCAGATCACCCGACGCAAGTGGGAGCAGAGTG-GTGCTG--CAGAGCATTACAA

Q1 GGCAGCTCAGGAAACCCGACGCAAGTGGGAGCAGGCTG-GTGCTG--CTGAGAAACACAG

Q2 GGCAGCACTGAAGACCCGAAGCAAGTTGGAGCAGGCTG-GTCTTG--CAGAGAAGCGCAG

T23 AGCCTCACAGATCTCTAAGCACAAGTCAGAGGCAGTCG-ATGAGG--CCCACCAACAGAG

T11 AGCCTCGCATATCTCTAAGTGCAAGTCAGAGGCGGTCG-ATGAGG--CCCACCAACAGAG

T22 CACTGTAC---------------------------CTC-ACATCT--CTCAGGA------

T10 CACTGTAC---------------------------CTC-AGATCT--CTCAGCA------

M5 AGCTGCGCAGATCACGCTGCGCAGGTGGGAGAAGTCTG-GTGTCT--CTGAGCAAAGGCA

T15 AGTGGCTCAGATCACTCAACACAAATGGGAGGAGGCTG-GAGTGG--CAGAGGAATACAA

T9 AGTGGCTCAGATCACTCAACACAAATGGGAGGAGGCTG-GAGTGG--CAGAGGAATACAA

M3 GGTGTCTCAGATCACGAAGAGCCGTTTAGAGTCAGCTG-GCACAG--CAGAGTACTTCAG

M2 GGCAGCTGAGATCACCCGTAGAGAGTGGGAGGCAACTA-ATGTGG--CTGAGTTCTGGAG

T3 GGCAGCAGAGATCACCAGAAGCAAGTGGGAGCAGGCTG-GTTATA--CAGAGCTCCGTAG

T13 GGTAGCGCTGATCACCCGACGCAAGTGGGAGCAGGCTG-GTGCTG--CAGAGCTATACAA

T7 GGCAGCGCTGATCACCCGACGCAAGTGGGAGCAGGCTG-GTGCTG--CAGAGCATTACAA

T5 GGCGGCGCAGATCACTCGACGCAGGTGGGAGCAGGGTG-GTGTTG--CAGAGACGCTCAG

Q5 GGAGGCACAGATCACCCGACGCAAGTGGGAGCAGGCTG-GTATTG--CAGAGAGAGACCG

Q7 GGCGGCACAGATCACCCGACGCAAGTGGGAGCAGGCTG-GTATTG--CAGAGAAAGACCA

Q6 GGCTGCACAGATCACCCTACACAAGTGGGAGCAGGCTG-GTATTG--CAGAGAGAGACCG

Q4 GGCGGCACAGATCACCCGACGCAAGTGGGAGCAGGCTG-GTGCTA--CAGAGAAAAGCAA

Q10 GGCGGCGATTATCACCCGACGCAAGTGGGAGCAGGCTG-GTGCTG--CAGAGTATTACAG

T24 CATTGCTGGCTACACCAAGCGCAAGTGGGAAAACAATGAGTACTGGCTGGAGAAAGAGAA

*

K1 GGCCTACCTGGAGGGCACGTGCGTGGAGTGGCTCCGCAGATACCTGAAGAACGGGAACGC

D1 GGCCTACCTGGAGGGCGAGTGCGTGGAGTGGCTCCACAGATACCTGAAGAACGGGAACGC

Q1 GACCTACCTGGAGGGCAAGTGCCTGATGTGGCTCCACAGATACCTGGAGCTCGGGAAGGA

Q2 GGCCTACCTGGAGGTCGATTGCTTGACGTGGCTCCGCAGATACCTGGAGCTCGGGAAGGA

T23 GGCATACCTGCAAGGTCCTTGCGTGGAGTGGCTCCATAGATACCTACGGCTGGGAAATGA

T11 GGCATACCTGCAAGGTCCTTGCGTGGAGTGGCTCCATACATACCTACAGCTGGGAAGTGA

T22 ------CCTGAAGAGCCACTGCTCAGATCTGCTGCAGAAATACCTGGAAAAAGGGAAGGA

T10 ------CCTGGAGGGCCACTGCTCAGATCTGCTGCAGAAATACCTGGAAAAAGGGAAGGA

M5 ATCCTTCTTGAAGGGCGAGTGTGTGGAGTCGCTCCGCACATACCTGGAGATAAGGAAGGA

T15 GGCCTACCTAGAGGGCACATGCGTGGAGACACTCCACAGATTGTTGG---------AGGA

T9 GGCCTACCTAGAGGGCACATGCGTGGAGACACTCCACAGATTGTTGG---------AGGA

M3 GGCCTATGTGGAGGGAGAGTGCCTGGAGCTGCTCCACAGATTCCTGCGGAATGGCAAGGA

M2 GGTCTACTTGGAGGGCCCTTGCATGGTGTGGCTCTTTAAATACCTGACAGTGGGAAATGA

T3 GACCTACTTGGAGGGCCCATGCAAGGATTCCCTGCTCAGATACCTGGAGAACAGAAAAAA

T13 GTTCTACCTGGAGGCCGAGTGCGTGGAGTGGCTCCGCAGATACCTGCAGCTCGGGAAGGA

T7 GGCCTACCTAGAGGGCGAGTGCGTGGAGTCGCTCCGCAGATACCTGCAGCTCGGGAAGGA

T5 GGCCTACCTGGAGGACCCGTGCCTGGAGTGGCTCCGCAGATACCTGGAGCTCGGGAAGGA

Q5 GGCCTACCTGGAGGTCAGG------------CTCCGCAAATACCTGCAGCTCGGGAAGGA

Q7 GGCCTACCTGGAGGGCACGTGCATGCAGTCGCTCCGCAGATACCTGCAGCTCGGGAAGGA

Q6 GGCCTACCTGGAGGGCGCTTGCGTGCAGTCGCTCCGCAGATACCTGCAGCTCCGGAAGGA

Q4 GGCCTACCTGGAGGGCGCGTGCGTGCAGTCCCTCCGCAGATACCTGGAGCTCGGGAAGGA

Q10 GGCCTACCTGGAGGCCGAGTGCGTGGAGTGGCTCCTCAGATACCTGGAGCTCGGGAAGGA

T24 GACCTACTTGGAGAAGGAGTGCATCCTATGGCTGCAGAGATACTTAACCATGGGAGGAAA

* * ** * ** *

K1 GACGCTGCTGCGCACAGATTCCCCAAAGGCCCATGTGACCCATCACAGCAGACCTGAAGA

D1 GACGCTGCTGCGCACAGATTCCCCAAAGGCACATGTGACCCATCACCCCAGATCTAAAGG

Q1 GATGCTGCTGCGCACAGACCCCCCAAAGGCACATGTGACTCATCACCCCAGATCTCAAGG

Q2 GACGCTGCTGCACACAGATCCCCCAAAGGCGCATGTGACCCATCACCCCAGTTCTCAAGG

T23 GACACTGCAGCGCTCAGACCCTCCAAAGGCACATGTGACCCATCACCCTAGATCTGAAGA

T11 GACACTGCTGCGCTCAGACCCTCCAAAGGCACATGTGACCCGTCACCCCAGACCTGAAGG

T22 GAGGCTGCTGCGTTCAGACCCTCCAAAGGCACATGTGACCCGTCACCCCAGACCTGAAGG

T10 GAGGCTGCTGCGTTCAGACCCCCCAAAGGCACATGTGACCCGTCACCCCAGACCTGAAGG

M5 GACTCTGCTAAGAACAGATCCTCCCAAGGCACATGTGACCCATCACCCCAGACCTGAAGG

T15 AGCTCAGCAACACTCAGACCCCCCAAATACACATGTGACCCGTCACCCCAGACCTGAAGG

T9 AGCTCAGCAACACTCAGACCCCCCAAATACACATGTGACCCGTCACCCCAGACCTGAAGG

M3 GATTCTTCAGCGAGCAGATCCCCCAAAGGCACATGTGGCCCATCACCCGAGACCCAAAGG

M2 GACTCTACTGCGCACAGAGCCCCCAAAGGCATATGTGACCCATCACCCCAGACCTGAAGG

T3 GACACAGGAGTGCACAGATCCTCCAAAAACACACGTGACCCATCACCCCAGACCTGAAGG

T13 GACGCTGCTGCGCACAGATCCCCCAAAGGCACATGTGACCCATCATCCCAGACCTGCAGG

T7 GACGCTGCTGCGCACAGATCCCCCAAAAGCCCATGTGACCCATCACCCCAGATCTGAAGG

T5 GACGCTGCTGCGCACAGATCCCCCAAAGGTACATGTGACCCATCATCCCAGATCTGAAGA

Q5 GACGCTGCTGCGCACAGATCCCCCAAAGACACATGTGACCCATCACCCCATATCTTATGA

Q7 GACGCTGCTGCGCACAGATCCCCCAAAGGCACATGTGACCCATCACCCCAGGTCTTATGG

Q6 GACGCTGCTGTGCACAGATCCCCCAAAGGCACATGTGACCCATCACCCCAGGTCTTATGG

Q4 GACGCTGCTGCGCACAGATCCTCCAAAGGCACATGTGACATGTCACCACAGATCTGACGG

Q10 GACGCTGCTGCGCACAGATCCCCCAAAGACACATGTGACCCATCACCCAGGATCTGAAGG

T24 GAACTTTACCCGAACTGACCCTCCAAAGACAACAGTGACTCATCAGTTCAAACCTAAAGA

* ** * ** ** *** * *** * *

K1 TAAAGTCACCCTGAGGTGCTGGGCCCTGGGCTTCTACCCTGCTGACATCACCCTGACCTG

D1 TGAAGTCACCCTGAGGTGCTGGGCCCTGGGCTTCTACCCTGCTGACATCACCCTGACCTG

Q1 TGATGTCACCCTGAGGTGCTGGGCCCTGGGCTTCTACCCTGCTGACATCACCCTGACCTG

Q2 TGATGTCACCCTGAGGTGCTGGGCCCTGGGCTTCTACCCTGCTGACATCACCCTGACCTG

T23 TGAAGTCACCCTGAGGTGCTGGGCCCTGGGCTTCTACCCTGCTGACATCACCCTGACCTG

T11 TGATGTCACCCTGAGGTGCTGGGCCCTGGGCTTCTATCCTGCTGACATCACCCTGACCTG

T22 TGATGTCACCCTGAGGTGTTGGGCCCTGGGCTTCTACCCTGCTGACATCACCCTGACCTG

T10 TGATGTCACCCTGAGGTGCTGGGCCCTGGGTTTCTACCCTGCTGACATCACCCTGACCTG

M5 TGAAGTCACCCTGAGGTGCTGGGCCCTGGGCTTCTACCCTGCTAACATCATCCTGACCTG

T15 TGATGTCACCCTGAGGTGCTGGGCCCTGGGCTTCTACCCTGCTGACATCACCCTGACCTG

T9 TGAAGTCACCCTGAGGTGCTGGGCCCTGGGCTTCTACCCTGCTGACATCACCCTGACCTG

M3 TGATGTTACCTTGAGGTGCTGGGCTCTGGGCTTCTACCCTGCTGACATCACCCTGACCTG

M2 TGATGTCACCCTGAGGTGCTGGGCCCTGGGTTTCTACCCTTCTGACATCATCATGATCTG

T3 TTATGTCACCCTGAGATGCTGGGCCCTGCGCTTCTACCCTGCTGACATCACCCTGACCTG

T13 TGATGTCACCCTGAGGTGCTGGGCCCTGGGCTTCTACCCTGCTGACATCACCCTGACCTG

T7 TGATGTCACCCTGAGGTGCTGGGCCCTGGGCTTCTACCCTGCTGACATCACCCTGACCTG

T5 TGATGTCACCCTGAGGTGCTGGGCCCTGGGTTTCTACCCTGCTGACATCACCCTGACCTG

Q5 TGCTGTCACCCTGAGGTGCTGGGCCCTGGGCTTCTACCCTGTTGACATCACCCTGACTTG

Q7 TGCTGTCACCCTGAGGTGCTGGGCCCTGGGCTTCTACCCTGCTGACATCACCCTGACTTG

Q6 TGCTGTCACCCTGAGGTGCTGGGCCCTGGGCTTCTACCCTGCTGACATCACCCTGACTTG

Q4 TGATGTCACCCTGAGGTGCTGGGCCCTGGGCTTCTACCCTGCTAACATCATCCTGACCTG

Q10 TGATGTCACCCTGAGGTGTTGGGCCCTGGGCTTCTACCCTGCTGACATCACCCTGACCTG

T24 AAACGTCACCCTGAGGTGCTGGGCCCTGGGCTTCTACCCTGCTGACATCACCCTGACCTG

** *** **** ** ***** *** * ***** *** * ****** * *** **

K1 GCAGTTGAATGGGGAGGAGCTGATCCAGGACATGGAGCTTGTGGAGACCAGGCCTGCAGG

D1 GCAGTTGAATGGGGAGGAGCTGACCCAGGACATGGAGCTTGTGGAGACCAGGCCTGCAGG

Q1 GCAGTTGAATGGGGAGGAGCTGACCCAGGACATGGAGCTTGTGGAGACCAGGCCTGCAGG

Q2 GCAGTTGAATGGGGAGGACCTGACCCAGGACATGGAGCTTGTGGAGACCAGGCCTTCAGG

T23 GCAGTTGAATGGGGAGGAGCTGACCCAGGACATGGAGCTTGTGGAGACCAGGCCTGCAGG

T11 GCAGTTGAATGGGGAGGAGCTGACCCAGGACATGGAGTTTGTGGAGACCAGGCCTGCAGG

T22 GCAGTTGAATGGGGAGGAGCTGACCCAGGACATGGAGCTTGTGGAGACCAGGCCTGCAGG

T10 GCAGAAGGATGGGGAGGAGCTGACCCAAGAAGTGGAGTTTGTGGAGACCAGGCCTGCAGG

M5 GCAGTGGGATGAGGAAGACCTGACTCAGGACATGGACCTCATTGAGACCAGACCTGCAGG

T15 GCAGTTGAATGGGGAGGAGCTGACCCAGGACATGGAGCTTGTGGAGACCAGGCCTGCAGG

T9 GCAGTTGAATGGGGAGGAGCTGACCCAGGACATGGAGCTTGTGGAGACCAGGCCTGCAGG

M3 GCAGAAGGATGAAGAGGACCTGACCCAGGACATGGAGCTTGTGGAAACGAGGCCTTCAGG

M2 GCAAAGAGATGGGGAGGACCAGACCCAGGACATGGATGTTATTGAGACCAGACCTGCAGG

T3 GCAGTTGAATGGGGAGGAATTGATTCAGGACACGGAGCTTGTGGAGACCAGGCCTGCAGG

T13 GCAGTTGAATGGGGAGGAGCTGACTCAGGACATGGAGCTTGTGGAGACCAGGCCTGCAGG

T7 GCAGTTGAATGGGGAGGAGCTGACCCAGGACATAGAGCTTGTGGAGACCAGGCCTGCAGG

T5 GCAGTTGAATGGGGAGGAGCTGACCCAGGACATGGAGCTTGTGGAGACCAGGCCTGCAGG

Q5 GCAGTTGAATGGGGAGGAGCTGACCCAGGACACGGAGCTTGTGGAGACCAGGCCTGCAGG

Q7 GCAGTTGAATGGGGAGGAGCTGACCCAGGACATGGAGCTTGTGGAGACCAGGCCTGCAGG

Q6 GCAGTTGAATGGGGAGGAGCTGACCCAGGACATGGAGCTTGTGGAGACCAGGCCTGCAGG

Q4 GCAGTTGAATGGGGAGGAGCTGACCCAGGACATGGAGCTTGTGGAGACCAGGCCTTCAGG

Q10 GCAGTTGAATGGGGAGGAGCTGACCCAGGACATGGAACTGGTGGAGACCAGGCCTGCAGG

T24 GCAGTTGAATGGAGAGGAGCTGACCCAGGACACGGAGCTTGTGGAGACGAGGCCTTCAGG

*** *** ** ** ** ** ** ** * * ** ** ** *** ****

K1 GGATGGAACCTTCCAGAAGTGGGCATCTGTGGTGGTGCCTCTTGGGAAGGAGCAGTATTA

D1 GGATGGAACCTTCCAGAAGTGGGCATCTGTGGTGGTGCCTCTTGGGAAGGAGCAGAATTA

Q1 GGATGGAACCTTCCAGAAGTGGGCATCTGTGGTGGTGCCTCTTGGGAAGGAGCAGAATTA

Q2 GGATGGAACCTTCCAGAAGTGGGCATCTGTGATGGTGCCTTTTGGGGAGGAGCCTAGATA

T23 GGATGGAACCTTCCAGAAGTGGGCAGCTGTCGTGGTGCCTCTTGGGAAGGAGCAGTATTA

T11 GGATGGAACCTTCCAGAAGTGGGCATCTGTGGTGGTGCCTCTTGGGAAGGAGCAGAATTA

T22 GGATGGAACCTTCCAGAAGTGGGCAGCTGTGGTGGTGCCTCTTGGGAAAGAGCAGAGTTA

T10 GGATGGAACCTTCCAGAAGTGGGCAGCTGTGGTGGTGCCTCTTGGAAAGGTGCAGAGTTA

M5 GGATGGGACCTTCCAGAAGTGGGCATCTGTGGTGGTACCTTCTGGAGAGGAACAGAGATA

T15 GGATGGAACCTTCCAGAAGTGGGCAGCTGTGGTGGTGCCTCTTGGGAAGGAGCAGAATTA

T9 GGATGGAACCTTCCAGAAGTGGGCAGCTGTCGTGGTGCCTTTTGGGGAGGAGCCTAGATA

M3 GGATGGAACCTTCCAGAAGTGGGCAGCTGTGGTGGTGCCTTCTGGAGAGGAGCAGAGATA

M2 GGATGGAACCTTCCAGAAGTGGGTAGCTGTGGTGGTGCCTTCTGGGAAGGAGCAGAATTA

T3 GGATGGAACCTTCCAGAAGTGGGCAGCTGTGGTGGTGCCGCTTGGGAAGGAGCAGAAATA

T13 GGATGGAACCTTCTAG--------------------------------------------

T7 GGATGGAACCTTCCAGAAGTGGGCAGCTGTGGTGGTGCCTCTTGGGAAGGAGCAGAATTA

T5 GGATGGAACCTTCCAGAAGTGGGCATCTGTGGTGGTGCCTCTTGGAAAGGAGCAGAATTA

Q5 GGATGGAACCTTCCAGAAGTGGGCAGCTGTGATGGTGCCTTTTGGGGAGGAGCAGAATTA

Q7 GGATGGAACCTTCCAGAAGTGGGCATCTGTGGTGGTGCCTCTTGGGAAGGAGCAGAATTA

Q6 GGATGGAACCTTCCAGAAGTGGGCATCTGTGGTGGTGCCTCTTGGGAAGGAGCAGAATTA

Q4 GGATGGAACCTTCCAGAAGTGGGCATCTGTGGTGGTGCCTCTTGGGAAGGAGCAGAATTA

Q10 GGATGGAACCTTCCAGAAGTGGGCATCTGTGGTGGTGCCTCTTGGGAAGGAGCAGAATTA

T24 GGATGGGACATTCCAGAAGTGGGCAGCTGTGGTGGTGCCTTCTGGAGAGGAGCAGAGATA

****** ** *** **

K1 CACATGCCATGTGTACCATCAGGGGCTGCCTGAGCCCCTCACCCTGAGATGGGA------

D1 CACATGCCGTGTGTACCATGAGGGGCTGCCTGAGCCCCTCACCCTGAGATGGGA------

Q1 CACATGCCATGTGTACCATGAGGGGCTGCCTGAGCCCCTCACCCTGAGATGGGA------

Q2 CACATGCCATGTGGAACATGAGGGGCTGCCTGAGCCCCTCACCCTGAGATGGGA------

T23 CACATGCCATGTGTACCATGAGGGGCTGCCTGAGCCCCTCACCCTGAGATGGGA------

T11 CACATGCCATGTGTACCATGAGGGGCTGCCTGAGCCCCTCACCCTGAGATGGGA------

T22 CACATGCCATGTGTACCATGAGGGGCTGCCTGAGCCCCTCATCCTGAGATGGGA------

T10 CACGTGCCATGTGGACCATGAGGGGCTGCCTGAGCCCCTCACCCTGAGATGGGA------

M5 CACATGTCATGTGCAACATGAGGGGCTAACCCAGCCCCTTGTTCTAAAATGGGA------

T15 CACATGCCATGTGCACCATGAGGGGCTGCCTGAGCCACTCACCCTGAGATGGGA------

T9 CACATGCCATGTGTACCATTAG--------------------------------------

M3 CACATGTTATGTGCACCATGAGGGGCTAACCGAGCCCCTTGCCCTGAAATGGGGAA----

M2 CACATGCCATGTGGCTCATGAGGGACTGCCTGAGCCCCTCACCCTGAGATGGAGTA----

T3 CACATGTCATGTGTACCATGAGGGGCTGCCTGAGCCTCTCACCCTGAGATGGGA------

T13 ------------------------------------------------------------

T7 CACATGCCATGTGGAACATGAGGGGCTGCCTGAGCACCTCACCCTGAGATGGGA------

T5 CACATGCCATGTGTACCATGAGGGGCTGCCTGAGCCCCTCACCCTGAGATGGGA------

Q5 CACATGCCATGTGCACCATGAGGGGCTGCCTGAGCCCCTCACCCTGAGATGGG-------

Q7 CACATGCCATGTGAACCATGAGGGGCTGCCTGAGCCCCTTACCCTGAGATGGGGGAGATG

Q6 CACATGCCATGTGAACCATGAGGGGCTGCCTGAGCCTCTCACCCTGAGATGGG-------

Q4 CACATGCCATGTGCACCATGAGGGGCTGCCTGAGCCCCTCACCCTGAGATGGGA------

Q10 CACATGCCATGTGTACCATGAGGGGCTGCCTGAGCCCCTCACCCTGAGATGGGA------

T24 CACATGTCATGTGCAACATGAGGCACTAACGCAGCCCCTTGTTCTGAAATGGGA------

K1 ---GCCTCCTCCATCCACTGTCTCCAACATGGCGACCGTTGCTGT-TCTGGTTGT--CCT

D1 ---GCCTCCTCCGTCCACTGACTCTTACATGGTGATCGTTGCTGT-TCTGGGTGT--CCT

Q1 ---GCCTCCTCCATACACTGTTTCCAACATGGTAATCATAGCTGT-TCTGGTTGT--CCT

Q2 ---GCCTCCTCCATCCACTGACTCTTACATGGTGATCATTGCTGT-TCTGGTTGT--CCT

T23 ---GCCTCCTCCATCCACTGTCTCCAACATGGTAATCATAGCTGT-TCTGGTTGT--CCT

T11 ---GCCTCCTCCATCCACTGTCTCCAACATGGTAATCATTGCTGT-TCTGGTTGT--CCT

T22 ---GCCTGCATGGTACCAAAAGCCTTGGATTTGGATTGTTGCCATGGTTTTCATT--TTG

T10 ---GCCTGCATGGTACCAAAAGCCTTGGATTTGGATTGTTGCCACGGTTTTTTCC--ATT

M5 ---CCCTTCTAAGCACACCATTCCCATCATGGGAATCACTGT--TGGCCTGCTTC--TCT

T15 ---GCCTCTTCCATCCACCGACTCTAACATGGTAATCATTGCT-GCTCTGGTTGT--CTT

T9 ------------------------------------------------------------

M3 --GATCGTCTCAATCATCTGTCGTCATCATGGTCATCGTTGC--TAGTCTGGTTC--TCC

M2 --GACCTCCTCAGTCTTTCATTTTCATCATAATAGTTGCTGT--TGGCCTGGTTC--TCC

T3 ---GCCTCCTCAGACCAGTATGCCCAACAGGACCACTGTT-------CGTGCTCT--CCT

T13 ------------------------------------------------------------

T7 ---GCCTCCTCCATCCACGGACTCCAACATGGTAATCATGGCTGT-TCTTGTTGT--CCT

T5 ---GCCTCGTCCATCCAGGGACTCCAACATGGTAATCATAGCTGT-TCTGGTTGT--CCT

Q5 --AGCCTCCTCCATACACTGTCTCCAACATGGCGACCATTGCTGT-TGTGGTTGA--CCT

Q7 GGAGCCTCCTCCATACACTGTCTCCAACATGGCGACCATTGCTGT-TGTGGTTGA--CCT

Q6 --AGCCTCCTCCATCCACTGTCTCCAACATGGCGAACGTAGCTAT-TCTGGTTGT--CCT

Q4 ---GCCTCCTCCATCCACTGTCTCCAACATGGCGAACGTAGCTGT-TCTGGTTGT--CCT

Q10 ---ACCTCCTCCTTCCACTGACTCTATCATGTCACACATTG------CTGATCTG--CTG

T24 ---GCCTCTTCAGCTCACCACGCCCACGACAGGAGTATATGCTAGGGGAAGCTGTAGTCC

K1 TGGAGCTGCAAT------------------AGTCACTGGAGCTGTGGTGGCTTTTGTGAT

D1 TGGAGCTATGGC------------------CATCATTGGAGCTGTGGTGGCTTTTGTGAT

Q1 TGGAGCTGTGATAGTCATTGGAGCTGTGGTCATCATTGGAGTTATGGTGTCTTTTGTGAT

Q2 TGGAGCTGTGAT------------------CATCATTGGAGCTGTGGTGGCTTTTGTGAT

T23 TGGAGCTGTGAT------------------CATCCTTGGAGCTGTGGTGGCTTTTGTGAT

T11 TGGAGCTGTGAT------------------AGTCATTGTTGCTGTGGTGGCTTTTGTAAT

T22 TTCATCATTTGT------------------CTCTGTGTGGTTTGCATATGCATGAAGAAG

T10 TTGCTCATTTGT------------------CTCTGTGTGGCTCGCAGACCCATGAAGAAG

M5 TTGG--AGTTGT------------------GTTTACTGGAGCTGTGGTTGCC------AT

T15 TGGAGCTGTGAT------------------CATCATTGGAGCTGTGGTGGCTTTTATGAT

T9 ------------------------------------------------------------

M3 TGGG--AGGTGT------------------GATCACTATTGTTGTGGTGTGCAAGAGGAG

M2 TGGG--AGCTTC------------------AGTGGCTACTCTTGTCATGTGGAAGAAGAG

T3 TGGAGCTATGAT------------------CATCTTAGGTTTTATGAGCGGAAGTGTTAT

T13 ------------------------------------------------------------

T7 TGGAGCTGTGAT------------------CATCATTGGAGCTATGGTATCTTTTGTGTT

T5 TGGAGTTGTGAT------------------CATCATTGGAGCTATGGTGCCTTTTGTGTT

Q5 TGGAGCTGTGGC------------------CATCATTGGAGCTGTGGTGGCTTTTGTGAT

Q7 TGGAGCTGTGGC------------------CATCATTGGAGCTGTGGTGGCTTTTGTGAT

Q6 TGTAGCT-TGGC------------------CATCATTGGAGCTGTGGTGGATTTTGTGA-

Q4 TGGAGCT-TGGC------------------CATCATTGCAGCTGTGGTGGCTTTTGTGA-

Q10 TGGC--------------------------CATCATTAAAGCTCTGGTGGTATTTGTGA-

T24 TCAAGCAACCTTGCTGAGTG---------TCCTTGCGTTTCCTTTATTTGGCATAGTACT

K1 GAAGATGAGAAGGAGAAACACAGCTTTTCTTCTCACAGGTGGAAAAGGAGGGGACTATGC

D1 GAAGA---GAAGGAGAAACAC---------------AGGTGGAAAAGGAGGGGACTATGC

Q1 GAAGA---GGAGGAGAAACAA---------------AGGTGGACAAGGAGAGGACTGTGC

Q2 GAAAA---GAGGGAGAAACAC---------------AGGTGGAAAAGTAAGAGACTACGC

T23 GAAG-AGGAGGAGACACATAGGTGTAAAAGGATGCTATGCTCATGTTCTAGGCAGCAAGA

T11 GAAG-AGGAGGAAA-ACACAGG---------ATGCTATGCTCATGTTCTAG---------

T22 AATGCAGGTGGGAGAGGA-AGGCGTGACACCCAAGAAGCAGGCAGAGACAGTCCCCAAGA

T10 AATGCAGGTGGGAGAGGA-AGGCGTGACACCCAAGAAGCAGGCAGAGACAGTCCCCAAGA

M5 AGTG-ATGAGGAAGAGGA-AAGGTTTTCAGATAATAATT--CTAAAAACATTCCTGAAGG

T15 GAAGAGGAGAAACACAGG-TGGAAAAGGAGGGGTCTATTGTTGGGAGCCGCGCCCACATT

T9 ------------------------------------------------------------

M3 ---GGGTGCAGGTAAGCA-A----------------------------------------

M2 CTCGGGTGGGGAAAGGGG-CAGCCTCTGA-------------------------------

T3 GATGTGGATGAGAAAGAACAATGGTGGAAACGGAGACGATAACACTGCTGCATATCAGAA

T13 ------------------------------------------------------------

T7 GAAGAGCAGGAGAACAATAGGTGGAAAAGGAGGGGACTATGCTCCGGCTGGAGGAAGCAA

T5 GAAGAGCAGGAGAAAAATAGGTGGAAAAGAAGAGAACTATGCTCTGGCTGGAGGCAGCAA

Q5 GAATAGGAGGTGA-----------------------------------------------

Q7 GAATAGGAGGTGA-----------------------------------------------

Q6 ------------------------------------------------------------

Q4 ------------------------------------------------------------

Q10 ------------------------------------------------------------

T24 TGTGTTTGGACTTACCCGCTACAAAACATGGTCAGAAGAAAGAACTGGCCTGCTCCATCT

K1 TCTGGCTCCAGGC---------TCCCAGACCTCTGATCTGTCTCTCCCAGA-TTGTAAAG

D1 TCTGGCTCCAGGC---------TCCCAGAGCTCTGAAATGTCTCTCCGAGA-TTGTAAAG

Q1 TCTGGCTCCAAGCAGGGACAGCGCCCAGAGCTCTGATATCTCTCTCCTAGA-TTGTAAAG

Q2 TCAAGATCCAGGCAGGGACAGCCCCCAGAGCTCTGATATCTCTCTCCTAGAATTGTAA--

T23 GCTTCCAGACCTCTGACTGGCCTCAGAAGGCATGA-------------------------

T11 ------------------------------------------------------------

T22 CTCTAGCA-AGACTGTTGTGGATGATGAGGAGATGGGGGTTTGCTTTTGGAAGATTAAGT

T10 CTCTAGCA-AGACTGTTGTGGATGATGAGGAGATGGGGGTTTGCTTTTGGAAGATTAAGT

M5 GCTGA-------------------------------------------------------

T15 CGCCGTTACAAGATGGCGCTGACAGCTGTGTTCTAAGTGGTAAACAAATAATCTGCGCAT

T9 ------------------------------------------------------------

M3 ------------------------------------------------------------

M2 ------------------------------------------------------------

T3 TGAGAGGGAACACTTGTCCCTGAGCCCTCGGGCTGAATCTGAGGCACTCGGGGTGGAAGC

T13 ------------------------------------------------------------

T7 AGGTGGCCAGGGCTCTGACTTGTTTCTGGGGGCCTTCAAAGCTATCCTTCCTGGTCCTGC

T5 CAGTGTCCAGGGCTCTGCCTTGTTTCTGGAGGCCTTCA--GCCATCCTTCCTGA------

Q5 ------------------------------------------------------------

Q7 ------------------------------------------------------------

Q6 ------------------------------------------------------------

Q4 ------------------------------------------------------------

Q10 ------------------------------------------------------------

T24 GTTCCTGAAGAAGAGAGGCTCTAGGACAGGACCCAGGGACAATGTAAATAGTTTACAGCT

K1 TGATGGTTCATGACCCTCATTCTCTAGCGTGA----------------------------

D1 TGATGGTTCATGACTCTCATTCTCTAGCGTGA----------------------------

Q1 CATGA-------------------------------------------------------

Q2 ------------------------------------------------------------

T23 ------------------------------------------------------------

T11 ------------------------------------------------------------

T22 CCTGTAAAACTTGTCTAGGCCACTCCCCAGGAACTTCAGTTGGCGAGTCTTTACTGTCAC

T10 ACTGTAAGACTTGTCTAGGCCACTCCCCAGGAACTTCAGTTGGCGAGTCTTTACTGTCAC

M5 ------------------------------------------------------------

T15 GTGCCAAGGGTATCTTATGA----------------------------------------

T9 ------------------------------------------------------------

M3 ------------------------------------------------------------

M2 ------------------------------------------------------------

T3 TGGGATGAAGGATCTTCCTTCTGCCCCACCATTGGTCTCCTGA-----------------

T13 ------------------------------------------------------------

T7 AGGCACTAGGGGACATCTGCATCCTGACAGCTCCATGCTGCCCTGA--------------

T5 ------------------------------------------------------------

Q5 ------------------------------------------------------------

Q7 ------------------------------------------------------------

Q6 ------------------------------------------------------------

Q4 ------------------------------------------------------------

Q10 ------------------------------------------------------------

T24 CAAGGCAGACCTGGCCATGATCCTCAGAGGACACAGGTTTCAGTGA--------------

K1 -----

D1 -----

Q1 -----

Q2 -----

T23 -----

T11 -----

T22 CTTGA

T10 CTTGA

M5 -----

T15 -----

T9 -----

M3 -----

M2 -----

T3 -----

T13 -----

T7 -----

T5 -----

Q5 -----

Q7 -----

Q6 -----

Q4 -----

Q10 -----

T24 -----
